# Diesel exhaust particles alter the profile and function of the gut microbiota upon subchronic oral administration in mice

**DOI:** 10.1186/s12989-021-00400-7

**Published:** 2021-02-09

**Authors:** Sybille van den Brule, Margaux Rappe, Jérôme Ambroise, Caroline Bouzin, Chantal Dessy, Adrien Paquot, Giulio G. Muccioli, Dominique Lison

**Affiliations:** 1 Louvain centre for Toxicology and Applied Pharmacology, Institut de Recherche Expérimentale et Clinique, UCLouvain, Brussels, Belgium; 2 Centre de Technologies Moléculaires Appliquées, Institut de Recherche Expérimentale et Clinique, UCLouvain, Brussels, Belgium; 3 IREC Imaging Platform (2IP), Institut de Recherche Expérimentale et Clinique, UCLouvain, Brussels, Belgium; 4 Pole of Pharmacology and Therapeutics, Institut de Recherche Expérimentale et Clinique, UCLouvain and Cliniques Universitaires Saint-Luc, Brussels, Belgium; 5 Bioanalysis and Pharmacology of Bioactive Lipids Research Group, Louvain Drug Research Institute, UCLouvain, Brussels, Belgium

**Keywords:** Air pollution, Particles, Cardiovascular diseases, Metabolic diseases, Short-chain fatty acids, Atherosclerosis, ApoE

## Abstract

**Background:**

Ambient air pollution by particulate matters, including diesel exhaust particles (DEP), is a major cause of cardiovascular and metabolic mortality worldwide. The mechanisms by which DEP cause these adverse outcomes are not completely understood. Because the gut microbiota controls cardiovascular and metabolic health, we hypothesized that the fraction of inhaled DEP which reach the gut after mucociliary clearance and swallowing might induce gut dysbiosis and, in turn, contribute to aggravate or induce cardiovascular and metabolic diseases.

**Results:**

Female *ApoE*^−/−^ mice fed a Western diet, and wild-type (C57Bl/6) mice fed standard diet were gavaged with DEP (SRM2975) doses corresponding to mucociliary clearance from inhalation exposure (200 or 1000 ng/day, 3 times a week for 3 months; and 40, 200 or 1000 ng/day, 3 times a week for 6 months, respectively). No mortality, overt systemic or digestive toxicity was observed. A dose-dependent alteration of the gut microbiota was recorded in both strains. In *ApoE*^−/−^, β-diversity was modified by DEP, but no significant modification of the relative abundance of the phyla, families or genera was identified. In C57BL/6 mice, DEP reduced α-diversity (Shannon and Simpson indices), and modified β-diversity, including a reduction of the Proteobacteria and Patescibacteria phyla, and an increase of the Campylobacterota phylum. In both mouse models, perturbation of the gut microbiota composition was associated with a dose-dependent reduction of bacterial short chain fatty acids (butyrate and propionate) in cecal content. However, DEP ingestion did not aggravate (*ApoE*^−/−^), or induce (C57BL/6 mice) atherosclerotic plaques, and no metabolic alteration (glucose tolerance, resistance to insulin, or lipidemia) was recorded.

**Conclusions:**

We show here that oral exposure to DEP, at doses relevant for human health, changes the composition and function of the gut microbiota. These modifications were, however, not translated into ultimate atherosclerotic or metabolic outcomes.

**Supplementary Information:**

The online version contains supplementary material available at 10.1186/s12989-021-00400-7.

## Background

Ambient air pollution is a leading cause of mortality in the world [1], notably by cardiovascular (CV) diseases such as atherosclerosis, ischemic cardiopathy, and stroke [2–4]. Ambient air pollutants comprise gaseous or volatile components, and particulate matters (PM). The CV toxicity of ambient air pollution is mainly linked to PM components. Diesel exhaust particles (DEP) compose approximately 20% of the ambient PM, and belong to the fine (PM_2.5_) and ultrafine particle fractions (PM_0.1_). DEP exposure can impair endothelial and fibrinolytic functions, promote blood thrombogenicity and exacerbate cardiac ischemia in humans [5]. DEP inhalation aggravates experimental atherosclerosis in *ApoE*^−/−^ mice fed a lipid-rich diet [6]. The metabolic syndrome, which includes obesity, arterial hypertension, elevated triglycerides (TG) and cholesterol, and perturbation of glucose homeostasis, is also associated with air pollution and PM [7, 8]. PM_2.5_ inhalation can induce insulin resistance, adipose tissue inflammation, and diabetes in mice [9, 10].

The mechanisms by which ambient air pollutants cause cardio-metabolic (CM) toxicity are not completely understood, and may include pulmonary, systemic inflammation and oxidative stress, alteration of the autonomic CV regulation, and increased thrombogenicity caused in part by systemically translocated particles [2, 3]. After PM inhalation, a significant fraction of the particles deposited in the respiratory tract can also reach the gastrointestinal tract after mucociliary clearance [11]. This swallowed fraction of inhaled particles might interact with the gut microbiota (GM).

Alterations of the GM (dysbiosis) are associated with the development of various human diseases, including atherosclerosis, heart failure, obesity and type 2 diabetes [12, 13]. Modifications of the GM composition are associated with atherosclerotic diseases [14]. In experimental animals, several bacterial species can prevent the progression of atherosclerotic plaques [15]. Bacterial metabolites of the GM, including short chain fatty acids (SCFA), play crucial roles in the host metabolism and immunological processes. SCFA, mainly butyrate, acetate, and propionate, are produced in the cecum and the proximal colon, principally through the fermentation of dietary fibers. SCFA regulate energy homeostasis, insulin sensitivity, and glucose and lipid metabolism. Increased SCFA production is associated with a reduced risk of CV and metabolic diseases [16, 17].

Environmental chemicals shape the GM, and can induce gut dysbiosis [18–20]. Some studies have investigated the impact of air pollution particles on the GM, upon inhalation or oral exposure of wild-type (w.t.) animals or in disease models [21]. Inhalation of concentrated PM, PM_2.5_ or DEP in w.t. mice modified the richness and the composition of the GM, as well as glucose homeostasis or colon integrity [22–24]. In w.t., *IL-10*-, or low-density lipoprotein receptor (*Ldlr*)-deficient mice, oral exposure to PM_10_ or PM_0.1_ induced or aggravated intestinal inflammation, and altered the composition of the GM [25–27]. Gavage of *Ldlr*^−/−^ mice with PM_0.1_ also increased cecal cholesterol content and the plasma concentration of atherogenic lipids [27]. These studies were, however, conducted with a single and/or very high dose(s) of questionable relevance for human health, over a generally short period. Whether the alterations of the GM recorded after oral PM exposure are associated with CV or CM toxicity has not been investigated.

Here, we explore the possible contribution of the GM to mediate the CM toxicity of inhaled PM. We tested this hypothesis by subchronically administering DEP by gavage, at doses corresponding to mucociliary clearance from inhalation exposure. Oral administration was selected to eliminate the contribution of other mechanisms involving the respiratory tract or systemic translocation that mediate CM toxicity of inhaled DEP. We used two experimental models, i.e. *ApoE*^−/−^ mice fed a Western diet and w.t. (C57BL/6) mice fed a normal diet, to evaluate the potential of DEP to aggravate and/or to induce CM effects, respectively.

## Results

### Local and systemic effects of oral DEP exposure

**Fig. 1:**
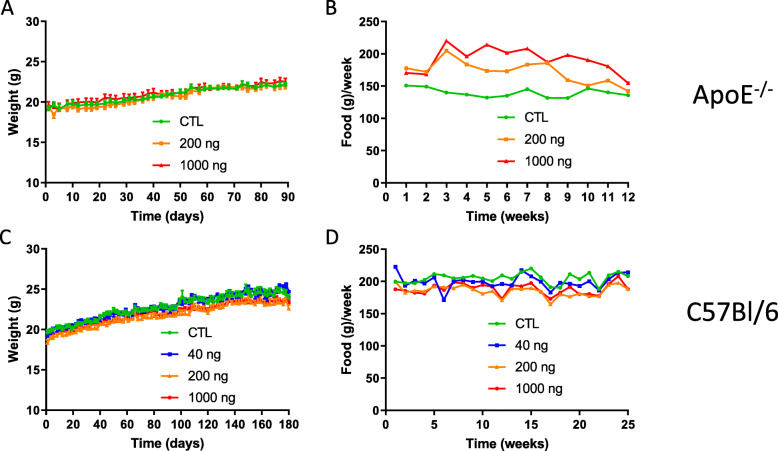
Effects of oral DEP exposure on mouse body weight and food consumption. Mice were gavaged 3x/week with PBS (CTL), 40, 200 or 1000 ng DEP/gavage. Body weight (**a**, **c**) was monitored 3x/week during the treatment period, and food consumption (**b**, **d**) was measured every week at the cage level for *ApoE*^−/−^ (**a**, **b**) and C57Bl/6 (**c**, **d**) mice

*ApoE*^−/−^ mice were gavaged with 0, 200 or 1000 ng DEP, 3 times a week for 3 months, and C57Bl/6 mice with 0, 40, 200 or 1000 ng DEP, 3 times a week for 6 months. These doses are representative of human inhalation exposure in urban environments (see Methods). No mortality was recorded during the course of the experiment. Body weight (b.w.) gain was constant and similar in all groups, suggesting the absence of severe toxicity (Fig. 1a and c). In *ApoE*^−/−^ mice, the animals treated with DEP ate more than controls (Fig. 1b). In a linear regression analysis, the dose-effect was significant (*p* < 0.05) but the interaction time*dose was not (*p* = 0.366), thus reflecting a parallel evolution over time of the food consumption among the different dose groups. In C57Bl/6 mice, food consumption did not differ among the treatment groups (Fig. 1d).

At the end of the exposure, blood cell counts were not altered by DEP exposure in *ApoE*^−/−^ mice. In C57Bl/6 mice, lymphocytes and monocytes were dose-dependently depleted (Additional file, Fig. S1), but red blood cell numbers were not affected (Additional file, Fig. S1I). Aspartate aminotransferase (AST) and alanine aminotransferase (ALT), measured in plasma to evaluate liver damage, were not affected by DEP exposure (Additional file, Fig. S2). Histological analyses did not reveal intestinal damage or structural alterations after DEP exposure. In both strains, ileal villi were well conserved, goblet cells were unaffected and the glycocalyx was intact in all experimental groups (Additional file, Figs. S3 and S4). Colonic tissue was not affected by the treatment (Additional file, Figs. S3 and S4). We concluded that oral DEP exposure did not induce overt local (digestive) or systemic toxicity, except a reduction of circulating lymphocytes and monocytes in C57Bl/6 mice.

### Oral DEP exposure alters the GM profile

The impact of DEP exposure on the composition of the GM was assessed by determining α- and β-diversity, and by comparing the relative abundance of phyla, families and genera in the different experimental groups. Richness and evenness of the microbial communities (within group diversity) were first analyzed by comparing three α-diversity indices. The simplest metric is the number of Amplicon Sequence Variants (ASVs) in a sample, which is also called richness. Simpson and Shannon indices account for richness and evenness in the populations. Evenness represents the degree to which individuals are split among ASVs. Beta-diversity accounts for the degree of variation in ASV composition across groups (between group diversity).

**Fig. 2:**
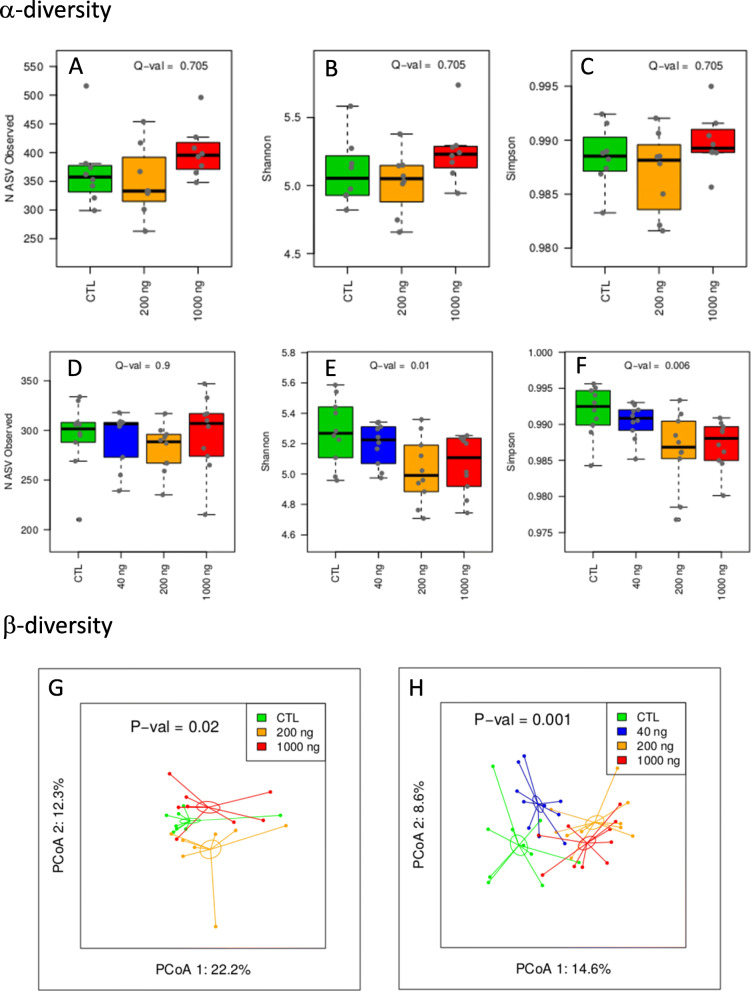
Oral DEP exposure alters the composition of the gut microbiota. Αlpha-diversity (A-F) and β-diversity (G, H) of the GM in *ApoE*^*−/−*^ (**a**-**c**, **g**) and C57Bl/6 (D-F, H) mice orally exposed to DEP. **a**-**f** α-diversities are presented as richness (number of ASVs, **a**, **d**), Shannon (**b**, **e**) and Simpson indices (**c**, **f**), Q-values are for linear trend tests, adjusted for FDR according to the Benjamini-Hochberg method, **g**, **h** Principal Coordinates Analysis (PCoA) is based on the weighted UniFrac distance matrix generated from all samples of each group. The dose-dependent effect was assessed by a permutational multivariate analysis of variance (ADONIS) of the weighted UniFrac distance matrix assuming a linear trend effect of the DEP dose on the microbial structure (**g**, *p* = 0.02 for ApoE−/−; **h**, *p* = 0.001 for C57Bl/6) (*n* = 8 for ApoE−/−; *n* = 10 for C57Bl/6).

In *ApoE*^−/−^ mice, DEP exposure did not significantly affect α-diversity (Fig. 2, A-C) but had a dose-dependent impact on α-diversity (linear dose-effect in ADONIS analysis; *p*-value, 0.02; Fig. 2g).

We did not detect significant modifications in the proportion of phyla, families or genera in *ApoE*^−/−^ mice, except a linear reduction of the relative abundance of the *Tannerellaceae* family (Additional file, Fig. S5, S6 and S7).

In C57Bl/6 mice, DEP exposure dose-dependently reduced α-diversity (Shannon and Simpson indices, Fig. 2d-f). Beta-diversity was dose-dependently modified by DEP treatment (linear dose effect in ADONIS analysis; *p*-value, 0.001; Fig. 2h). At the phylum level, DEP exposure dose-dependently reduced the relative abundance of Proteobacteria and Patescibacteria, and increased Campylobacteria and Cyanobacteria (Additional file, Fig. S8). At the family level, we recorded an expansion of *Helicobacteraceae*, and a reduction of *Sutterelaceae* (Additional file, Fig. S9). At the genus level, DEP exposure increased the relative abundance of *Roseburia* and *Helicobacter* and *Rikenellaceae_RC9_gut_group* (Additional file, Fig. S10).

### Oral DEP exposure disturbs the functions of the GM

**Fig. 3:**
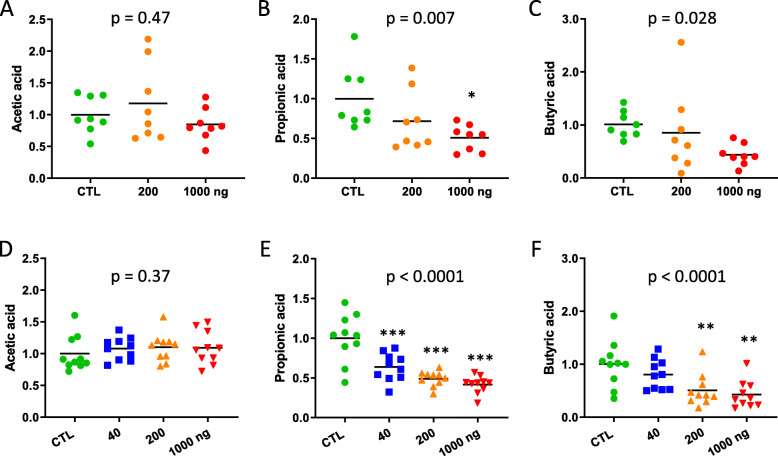
Effect of oral DEP exposure on cecal SCFA content Acetic (**a**, **d**), propionic (**b**, **e**) and butyric acid (**c**, **f**) were measured in the cecal content of *ApoE*^−/−^ (**a**-**c**) and C57Bl/6 (D-F) mice treated with PBS (CTL), 40, 200 or 1000 ng DEP/gavage. Data are expressed relative to the mean of CTL, and were analyzed by a one-way ANOVA followed by a Dunnett’s test (* *p* ≤ 0.05, ** *p* ≤ 0.01, *** *p* ≤ 0.001) and a test for linear trend (indicated *p* values) (*n* = 8 for *ApoE*^−/−^, *n* = 10 for C57Bl/6)

As an alteration of the GM composition can lead to a disturbed production of SCFA, acetic, propionic and butyric acids were quantified in the cecal contents. While the acetic acid content was not modified by DEP exposure in *ApoE*^−/−^ or in C57Bl/6 mice (Fig. 3a and d), propionic and butyric acids were dose-dependently reduced by oral DEP administration in both strains (Fig. 3b, c, e and f).

### Metabolic effects of oral DEP exposure

**Fig. 4:**
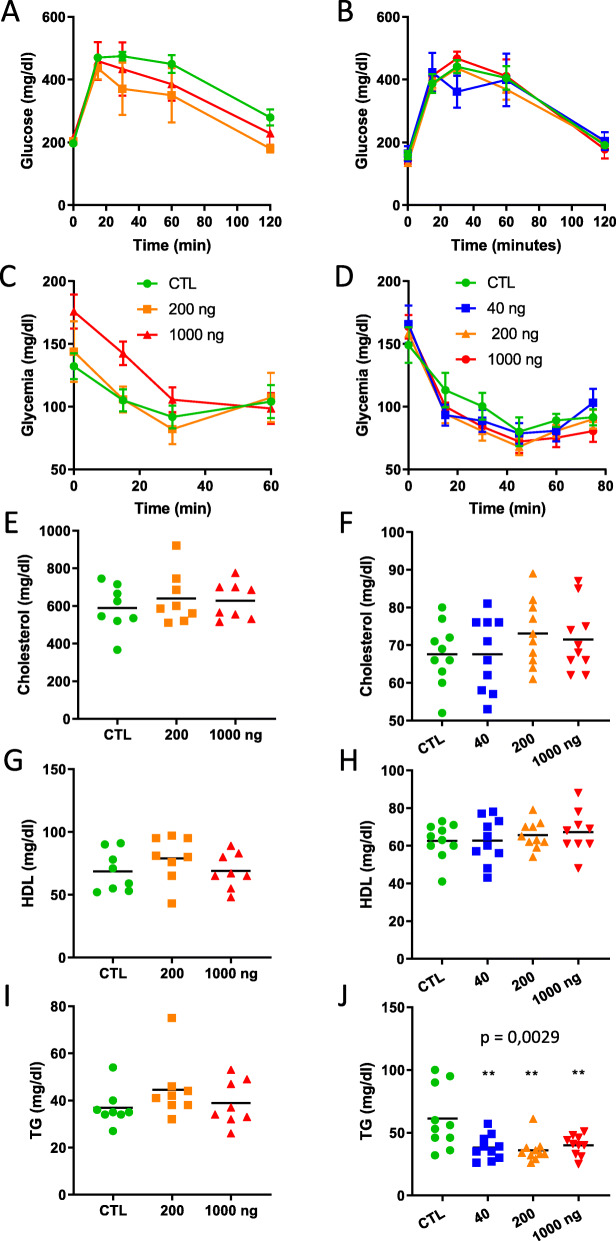
Metabolic responses after oral DEP exposure Metabolic effects were measured in *ApoE*^−/−^ (**a**, **c**, **e**, **g**, **i**) and C57Bl/6 (**b**, **d**, **f**, **h**, **j**) mice 3 and 6 months, respectively, after oral exposure to DEP. Glucose tolerance test (**a**, **b**; *n* = 4) and Insulin tolerance test (**c**, **d**; *n* = 6) responses were analyzed by a two-way ANOVA (dose*time interaction was not statistically significant). Plasma total cholesterol (**e**, **f**), HDL cholesterol (**g**, **h**) and triglycerides (TG) (**i**, **j**) were analyzed by a one-way ANOVA followed by a Dunnett’s test (** *p* ≤ 0.01) and a test for linear trend (indicated p values) (*n* = 8 for *ApoE*^−/−^, *n* = 10 for C57Bl/6)

Glucose homeostasis was evaluated by performing glucose and insulin tolerance tests, two weeks and one week before the end of exposure, respectively. None of these tests showed an impact of DEP exposure, neither in *ApoE*^−/−^ or in C57Bl/6 mice (Fig. 4a-d). As expected [28], blood cholesterol was much higher in *ApoE*^−/−^ than in C57Bl/6 mice, but DEP administration did not affect these levels, neither in *ApoE*^−/−^ or in C57Bl/6 mice (Fig. 4e, f). Blood high density lipoprotein (HDL) cholesterol levels were not modified by DEP exposure (Fig. 4g, h). Finally, triglycerides (TG) were not modified by DEP exposure in *ApoE*^−/−^ (Fig. 4i), but were strongly reduced by the treatment in C57Bl/6 mice (Fig. 4j).

### Cardiovascular effects of oral DEP exposure

**Fig. 5:**
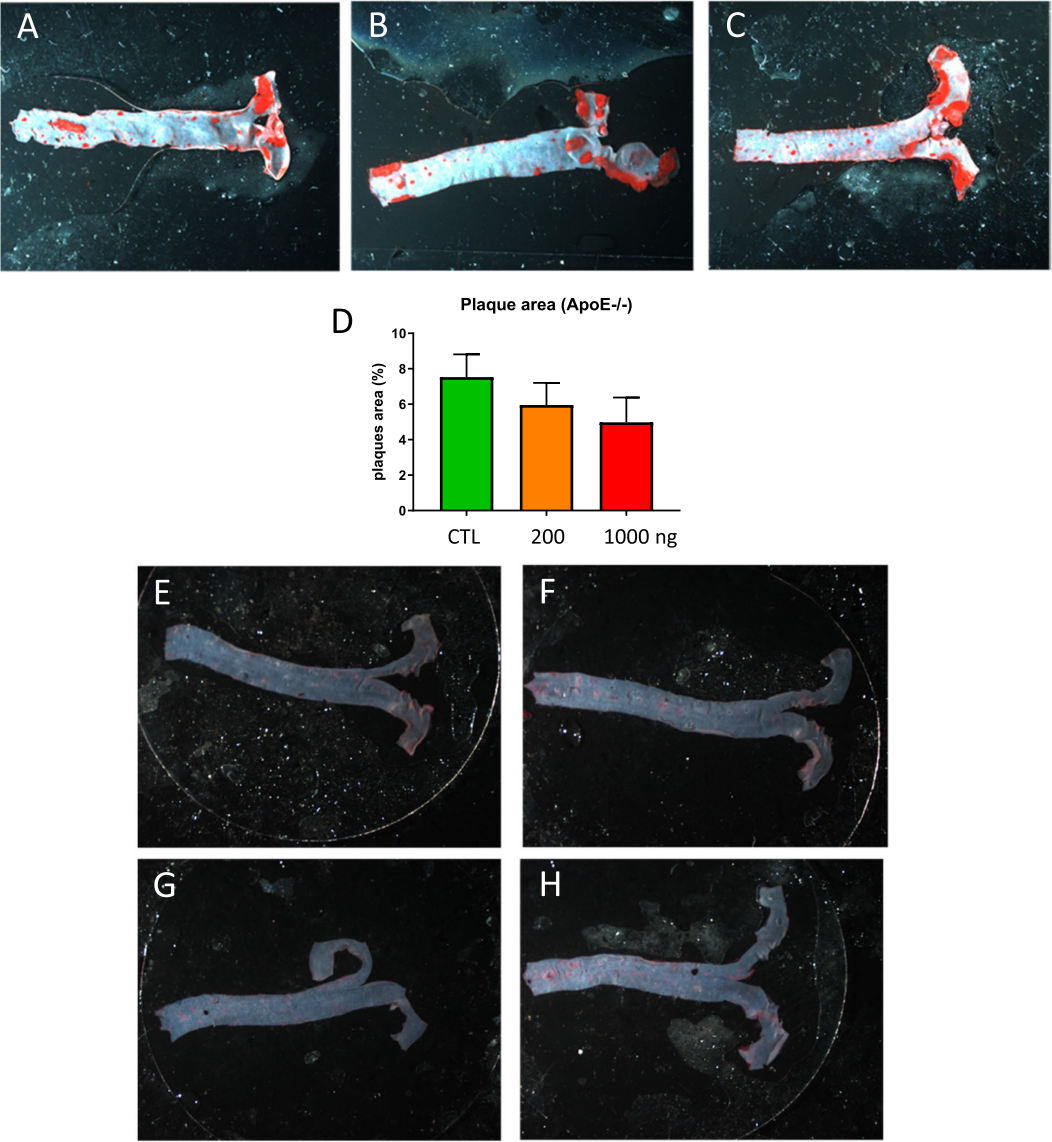
Atherosclerosis response after oral DEP exposure. Representative pictures of thoracic aortas stained with Oil red O from *ApoE*^−/−^ (**a**-**c**) and C57Bl/6 (E-H) mice: control (**a** and **e**) or treated with 40 (**f**), 200 (**b**, **g**) or 1000 ng/gavage (**c**, **h**). The % of plaque area (to the total aorta surface) is shown in (**d**) for *ApoE*^−/−^ mice. A one-way ANOVA did not detect significant difference among the groups (*n* = 8 *ApoE*^−/−^ mice)

Histological sections of abdominal aortas did not reveal an impact of DEP exposure on the arterial structure, neither in *ApoE*^−/−^ or in C57Bl/6 mice; the intima was intact and no inflammation was apparent (Additional file, Fig. S11). The Oil Red O staining of lipids and triglycerides in thoracic aortas is used to reveal atherosclerotic plaques. As expected [28], plaques were recorded in *ApoE*^−/−^ aortas (Fig. 5a-c). The number of plaques and the ratio of plaques area to total aorta surface were not modified by DEP exposure in *ApoE*^−/−^; DEP exposure even decreased the % of plaques area, but this effect was not significant (ANOVA, *p*-value 0.183; Fig. 5d). In C57Bl/6 mice, plaques were absent in controls and exposure to DEP did not significantly induce plaque formation (Fig. 5e-h).

## Discussion

We found that subchronic oral exposure to DEP modifies the composition and function of the GM in mice, at doses that can reach the digestive tract upon inhalation exposure. These GM alterations did, however, not appear to result in ultimate metabolic or CV toxicity. Thus, our initial hypothesis was only partly verified. This finding complements and extends the results of previous studies using inhalation or oral exposure to different ambient PM, at much higher dose levels than in the present study (Table 1).

**Table 1:** Experimental studies in mice investigating the effect of ambient particulate matters, or DEP on the gut microbiota, intestinal tract, liver, and/or glucose metabolism (M, males; F, females)

	Mouse model	Diet	Route	Material	Dose	Duration	Effect on			
							Gut	Liver	Glucos	GM
Tomaru et al. 2007 [29]	db/db, F	standard	intratracheal	DEP	100 μg/2 wk	12, 18 wk		+		
Sun et al. 2009 [10]	C57Bl/6, M	high fat	inhalation	PM_2.5_	72.7 μg/m^3^	24 wk			+	
Nemmar et al. 2013 [30]	streptozocin-induced diabetic TO, M	standard	Intratracheal	DEP	400 μg/kg b.w.	24 h		+		
Kish et al. 2013 [25]	w.t. and *IL-10*^−/−^ 129/Sv	standard	gavage	urban PM_10_	18 μg/g b.w./d 10–13 μg/g/d	7–14 d 35 d	+			+
Salim et al. 2014 [26]	*IL-10*^−/−^ 129/Sv, F	standard	food	urban PM_10_	9 μg/g food	10–20 wk	+			+
Li et al. 2017 [27]	*Ldlr*−/− C57Bl/6, M	Western diet	gavage	UFP	3 × 40 μg/wk					+
Xu et al. 2017 [31]	w.t. and *nrf2*^−/−^ C57Bl/6, M	standard	inhalation	PM_2.5_	64 μg/m^3^	12 wk		+	+	
Mutlu et al. 2018 [22]	C57Bl/6, M	standard	inhalation	PM	135 μg/m^3^	3 wk				+
Wang et al. 2018 [23]	C57Bl/6j, M	standard	inhalation	PM_2.5_	276 ± 170 μg/m^3^	1 y			+	+
Li et al. 2019 [32]	C57Bl/6, MF	standard	inhalation	DEP	300 μg/m^3^, 1 h/d	28 d	+			+
Fitch et al. 2020 [33]	*ApoE*^−/−^, M	high fat	inhalation	mixed diesel and gasoline exhaust dust	300 μg/m^3^	50 d	+			+
present study	C57Bl/6, F	standard	gavage	DEP	3x(40, 200 or 1000 ng)/wk	24 wk				+
	*ApoE*^−/−^, F	Western			3x(200 or 1000 ng)/wk	12 wk				

Similar to previous investigators [10, 25, 31], we did not record an alteration of animal b.w. after DEP exposure. While intestinal inflammation and injury, and modified gut permeability have been reported after PM administration [22, 25–27, 32, 33], our study did not reveal overt intestinal toxicity, based on histopathological analysis. We cannot, however, exclude subtler intestinal toxicity because we did not assess immunological cells and mediators, intestinal inflammation or permeability. We did not observe liver damage either, in contrast with previous reports [29–31]. Discrepancies between our results and previous studies can be attributed to differences in the animal models, the mode of exposure, the type of diet, and, mainly, the dose levels used (Table 1).

We recorded a dose-dependent reduction of blood lymphocytes and monocytes in C57Bl/6 mice after 24 weeks, but not in *ApoE*^−/−^ mice after 12 weeks exposure to DEP. This leukocyte reduction cannot result from hemodilution as red blood cell counts were not modified at the same time point. Immunosuppressive effects have already been reported in the spleen, Peyer’s patches and mesenteric lymph nodes after oral administration of SiO_2_ and TiO_2_ nanoparticles [34–36]. Further investigations will be needed to determine by which mechanism orally administered DEP can deplete circulating leukocytes.

Several studies have investigated the effects of PM or DEP on the GM after inhalation or oral exposure, leading to varying results (Table 2). These heterogeneous responses likely reflect the variety of materials tested, the differences in experimental models, routes of exposure, and dose levels (Table 1), but also the variety of samples analyzed for GM profiling (Table 2). We characterized the GM composition and SCFA production on cecal samples, which are considered optimal to investigate environmental effects on the GM [37]. Importantly, all previous studies were conducted with a single dose level of test material, which did not allow assessing dose-effect/response relationships. The characterization of the dose effect/response relationships is an integral element of any toxicology study, especially critical when investigating possible impacts on the GM. Indeed, when mice of the same experimental group are kept in the same cage during all the experiment, a “cage effect” can confound the GM variations [38], and possibly the downstream impacts, such as CV or metabolic toxicity. In the present study, we took two precautions to minimize a confounding by a “cage effect”. First, we homogenized the GM across all cages for 2 weeks before the start of the experiment [38]. Second, we used several dose levels (3 doses for ApoE^−/−^ and 4 doses for C57Bl/6 mice) in order to assess dose-effect relationships. We recorded dose-dependent effects on the GM composition and function, which allows to attribute the observed effects to DEP exposure. The modifications of the microbiota composition, at both the α- and β-diversity levels, were more marked in C57Bl/6 than in *ApoE*^−/−^ mice, probably reflecting the longer duration of exposure in C57Bl/6 than in *ApoE*^−/−^ mice. Another possible explanation of this difference might be that ApoE−/− mice are more resistant to change in the GM than C57Bl/6 mice. In C57Bl/6 mice, the most striking microbiota change induced by DEP was a dose-dependent increase in the relative proportion of *Helicobacter* (genus), also recorded at the family (*Helicobacteriaceae*) and phylum (Campylobacterota) levels. The *Helicobacter* genus encompasses more than 25 species, among which *H. pylori* is responsible for upper digestive diseases in humans. Non-*Helicobacter pylori* species are common in the digestive tract of rodents, including *H. hepaticus* and *H. bili.* Some of these *Helicobacter* species may be deleterious for the health, and their abundance has been correlated with obesity and psychiatric manifestations in mice [39]. Enrichment of *Helicobacter* was reported in the GM of mice treated with lactulose [40], high doses of mercury [41] or benzene [42]. In contrast, high altitude hypoxia can reduce the abundance of *Helicobacter* in mice [43]. We could not trace data on a possible association between *Helicobacter* and CV or metabolic diseases. The toxicological significance of the reduction in the relative abundance of Proteobacteria and Patescibacteria in C57Bl/6 mice exposed to DEP is not immediately evident. The Proteobacteria phylum normally represents a minor fraction of the mammalian GM, which includes several opportunistic pro-inflammatory bacteria (pathobionts) physiologically kept in check in low abundance. Gut dysbiosis is usually associated with an expansion of Patescibacteria [44], mainly reflecting intestinal epithelial dysfunction [45], rather than a reduction of this phylum [44]. An elevation of Proteobacteria was recently reported in mice fed with bisphenol-A [21]. Information on the role of the Patescibacteria superphylum component of the GM in mammalian (patho)physiology is not currently available.

**Table 2:** Main gut microbiota changes recorded after particulate matters, or DEP exposure in experimental mice

	Sample	α-diversity	Firmicutes	Bacteroidetes	Verrucomicrobia	Actinobacteria	Cyanobacteria	Lactobacillus	GM function	Toxicity
Kish et al. 2013 [25]	faecal		↑	↓	↑				↓SCFA^a^	
Li et al. 2017 [27]	caecal	↓	↓		↑	↓	↓		↑atherogenic lipids	
Mutlu et al. 2018 [22]	caecal	↑	↓	↑						
Wang et al. 2018 [23]	faecal	↓								Altered glucose metabolism
Li et al. 2019 [32]	faecal							↑		Colon inflammation, mucus depletion
Fitch et al. 2020 [33]	ileal tissue	↓	↓	↑						Altered intestinal integrity and inflammation

a: acetic, propionic and butyric acids

We observed dose-dependent reductions of propionic and butyric acids in cecal content both in C57Bl/6 and in ApoE−/− strains. In view of the major physiological role of butyric acid, including on the maintenance of intestinal barrier function, mucosal immunity, and CV and metabolic health [46–49], this observation suggested a deleterious health impact of orally administered DEP. A modification of the GM composition at the phylum level accompanied by modified fecal SCFA profiles has previously been observed in wild type and *IL-10* deficient mice gavaged with PM_10_ for 35 days [25]. These investigators also observed a decreased production of butyric acid. The exact microbial signature accounting for the reduction of propionic and butyric acid content in our study is not evident. Most butyrate-producing bacteria belong to the Firmicute phylum, which was (non-statistically significantly) reduced in C57Bl/6 mice (Fig. S8), not in ApoE−/− mice (Fig. S5). In contrast, propionate (and acetate)-producing bacteria typically belong to the Bacteroidetes phylum [50], which was (non-statistically significantly) increased in C57Bl/6 (Fig. S8), not in ApoE−/− mice (Fig. S5). Thus, there is no apparent correlation between phylum and SCFA changes, and it appears unlikely that SCFA changes can be explained by a phylum shift, most probably because subtler intra-phylum shifts exist between SCFA-producing and non-producing bacteria. Existing data on the potential metabolic toxicity of inhaled PM are unclear. Insulin resistance was recorded in C57Bl/6 mice exposed via inhalation to PM_2.5_ [10], and in obese rats that received PM_2.5_ via intratracheal instillation [51]. Gavage with UFP also increased the cecal cholesterol content in *Ldlr*-deficient mice [27]. Opposite results were observed in *Ldlr*-deficient mice exposed to UFP via inhalation [52]. We did not record deleterious effects on glucose homeostasis, blood cholesterol or total triglycerides neither in C57Bl/6 or *ApoE*^−/−^ mice.

Earlier studies have recorded an aggravation of atherosclerotic lesions upon inhalation exposure of genetically-deficient mice to DEP or other PM. In *Ldlr*^−/−^ mice, inhalation of UFP strongly increased the atherosclerotic lesions [52]. In *ApoE*^−/−^ mice fed a high fat diet, oropharyngeal aspiration of DEP (35 μg twice weekly for 4 weeks) increased atherosclerotic plaques, whereas DEP did not induce plaques in w.t. mice fed normal chow [6]. An exploratory study in *Ldlr*^−/−^ mice on high fat diet gavaged with UFP (40 μg/d, 3 days per week, for 10 weeks) suggested that oral exposure to PM could aggravate CV effects as assessed by circulating atherogenic lipids, but atherosclerotic plaques were not investigated [27]. In the present study, we examined, for the first time, the possible aggravation and/or development of atherosclerotic plaques, an ultimate manifestation of CV toxicity, after oral exposure to DEP. Although we recorded changes in the GM composition and SCFA production, notably a reduced production of butyrate which is essential for cardiometabolic health [16], we did not detect signs of CV toxicity. The alterations of the α- and β-diversity of GM recorded in C57Bl/6 mice treated with DEP were not associated with a consequent atherosclerotic response, which is consistent with previous findings in male C57Bl/6 mice treated twice weekly for 4 weeks with an oropharyngeal aspiration of 35 μg of DEP [6]. In *ApoE*^−/−^ mice, we observed atherosclerotic plaques after 3 months, but no significant aggravation upon DEP administration. Several factors might contribute to explain the absence of atherosclerotic effect of DEP in *ApoE*^−/−^ mice: (i) orally administered DEP may not significantly exacerbate the pathogenesis of atherosclerosis in *ApoE*^−/−^ mice fed a Western diet, (ii) the alterations of the GM recorded in the present study may not be qualitatively or quantitatively sufficient to accelerate atherosclerosis, (iii) the realistic, but relatively low doses administered to the mice may not accelerate atherosclerosis, (iv) the exposure durations (3 months) might be insufficient to aggravate the atherosclerotic process, and importantly (v) female mice may be less susceptible to atherosclerosis than male mice. Sexual hormones indeed play an important role in the development of CV diseases, explaining that estrogens protect non-menopausal females against CV diseases [53]. As the present study was conducted with female mice, we might need to verify our findings in male mice. Another limitation of the present study is that we cannot exclude other manifestations of CV toxicity than atherosclerosis, including alterations of the ECG, thrombogenesis, or myocardial disease.

## Conclusions

We found that subchronic oral DEP exposure of w.t. and *ApoE*^−/−^ female mice, at dose relevant for human health, altered the composition and function of the GM, but did not induce metabolic toxicity or atherosclerotic responses.

## Methods

### DEP and dose selection

The SRM2975 DEP standard was obtained from the National Institute of Standards and Technology (NIST, Gaithersburg, MD, USA). Particles were suspended in phosphate-buffered solution (PBS) at 1 mg/ml and sonicated 15 min at 40% of maximal power with a VC750 ultrasonic processor (Sonics & Materials, Newtown, USA) equipped with a 3 mm probe. Fresh suspensions were prepared for each gavage, and serially diluted in PBS to obtain the appropriate gavage doses (see below) in 200 μl. The hydrodynamic mean diameter (Z-average) was measured by dynamic light scattering (DLS) with a Zetasizer nano ZS (Malvern, Orsay, France) on a 5.15 μg SRM2975/ml suspensions in PBS after sonication (concentration of DEP in the suspension used to deliver 1000 ng/gavage). Figure S12 in additional file 1 depicts a mono-disperse size distribution for SRM2975 in PBS, and indicates a Z-average of 575 nm.

Mice received gavage doses realistic for inhalation human exposure to PM in urban environments, which typically ranges from 20 to 1000 μg/m^3^ at peak concentrations [25]. To estimate gavage doses, we assumed that an adult human inhales 10 m^3^ of outdoor air/day, that the epithelial surface area of the human lung surface is 5.10^4^ larger than that of the mouse, that 30% of inhaled PM deposits in the human respiratory tract, of which 60% is deposited in the upper airways and trachea-bronchial regions [54], and can be translocated to the digestive tract via mucociliary clearance and swallowing. Finally, mice were gavaged 3 times a week instead of every day of the week. Thus, human inhalation exposures to 20, 100 and 500 μg/m^3^ would approximately correspond to 40 (20*10*0.30*0.60*0.0005*7*0.33*10^3^), 200 and 1000 ng/gavage in mice, a dose range reasonably relevant for human health. These oral doses are conservative because DEP only represent a fraction of the ambient PM mass, and also peak instead of average inhalation values were considered for exposure estimates. In contrast, a daily inhalation volume of 10 m^3^ instead of 15 m^3^, which is usually considered for environmental exposures, may lead to underestimate the oral doses. Considering a mouse b.w. of 20 g, gavage doses of 40, 200 and 1000 ng/d correspond to 2, 10 and 50 ng/g b.w./d, which is 3 orders of magnitude lower than in earlier studies (Table 1).

### Animals and experimental design

Four week-old B6.129P2-APOE/J (*ApoE*^−/−^) and C57Bl/6JRj (C57Bl/6) female mice were obtained from Charles River (L’Arbresle, France) and Janvier-Labs (Saint Berthevin, France), respectively. The mice were housed in autoclaved air-filtered polycarbonate cages with conventional sawdust (Carfil, Oud-Turnhout, Belgium) in a controlled environment (22 °C, 55% relative humidity, 16-h light/8-h dark cycle, with acidified water and food ad libitum). At the age of 6 weeks, litters were mixed every day for two weeks to homogenize the baseline GM before exposure [55]. Mice were tagged individually to follow their body weight (3 times a week) and behavior. Food consumption was recorded once a week per cage.

*ApoE*−/− mice (*n* = 24) were fed a standard diet (Altromin, Carfil) until the age of 8 weeks and, thereafter, a Western-type diet (21% fat, 0.15% cholesterol and 19.5% casein, Altromin, Carfil) was provided to accelerate the development of atherosclerotic plaques. After GM homogenization, mice were randomly assigned to experimental groups (3 groups, one cage/dose group, *n* = 8). Mice were gavaged 3 times a week (Monday, Wednesday and Friday) for 3 months. This duration was selected based on significant mortality reported in this mouse strain fed a Western diet [56]. Mice were gavaged with a volume of 200 μl PBS containing 0 (control group, CTL), 20 or 1000 ng DEP.

C57Bl/6 mice (*n* = 40) were fed a standard diet during the whole experiment (before and during exposure). After GM homogenization, mice were randomly assigned to experimental groups (4 groups, one cage/dose group, *n* = 10). Mice were gavaged 3 times a week (Monday, Wednesday and Friday) for 6 months with a volume of 200 μl PBS containing 0 (control group, CTL), 40, 200 or 1000 ng DEP.

A glucose tolerance test (n = 4 per dose group) and an insulin tolerance test (*n* = 6 per dose group) were performed in both strains, respectively two and one week before the end of exposure according to Wang et al. [23]. At the end of the exposure period, mice were euthanized by an intra-muscular injection of 60 mg of pentobarbital, and cardiac blood was collected in EDTA tubes for blood cell counting (MS9–3, Melet Schloesing) and preparation of plasma. The abdominal aorta, the 3 distal centimeters of the ileum and the 3 proximal centimeters of the colon were excised, washed with PBS and stored in formaldehyde 4% for histological analysis. Hematoxylin-eosin (H&E) staining was performed on 5 μm thick sections of abdominal aorta, ileum and colon. The thoracic aorta was longitudinally open and transferred *en bloc* in formaldehyde 4% before staining with Oil red O. Cecal contents were collected and stored at − 20 °C until DNA extraction and quantification of SCFA.

All animal experiments were performed in accordance with local and institutional ethical guidelines. The experimental protocol was approved by the local committee for animal research at the UCLouvain, Comité d’Ethique pour l’Expérimentation Animale, Secteur des Sciences de la Santé, Brussels, Belgium (2018/UCL/MD/012).

### Biochemical analyses

AST/ALT, cholesterol, HDL cholesterol, triglycerides were analyzed in plasma by an Automated Clinical Chemistry Analyzer NX500 (Fujifilm, Tokyo, Japan).

### Detection of atherosclerotic plaques

After overnight incubation in formaldehyde, thoracic aortas were washed 5 min in PBS, incubated 5 min in isopropanol 60% and then stained 45 min at 37 °C under gentle agitation in Oil red O (stock solution 0.5% diluted 3:2 in distilled water). Aortas were finally washed 5 min in isopropanol 60% and 5 min in PBS. Pictures taken with a binocular were analyzed by ImageJ software (U.S. National Institutes of Health, Bethesda, USA) to determine the % of plaque area on the total surface of the aorta.

### Cecal DNA extraction and 16S rRNA gene sequencing

DNA was extracted with a QIAamp DNA Stool Mini kit according to manufacturer’s guidelines (Qiagen, Hilden, Germany), except DNA elution which was done with sterile PCR-grade water (Merck, Darmstadt, Germany). DNA concentrations were calculated on a Qubit Fluorometer (Invitrogen, Gent, Belgium) with a Qubit dsDNA HS assay kit. DNA samples were stored at − 20 °C until NGS analysis at MR DNA lab (Shallowater, USA) on a MiSeq system (Illumina, San Diego, USA). Specific primers of the 16S rRNA gene V4 variable region (forward GTGCCAGCMGCCGCGGTAA and reverse GGACTACHVGGGTWTCTAAT [57]) were used in a PCR with the HotStarTaq Plus Master Mix Kit (Qiagen) under the following conditions: 94 °C 3 min, (94 °C 30 s, 53 °C 40 s, 72 °C 1 min) × 28, 72 °C 5 min. PCR was performed in a single-step, meaning that a sample-specific barcode and a global primer was added to each amplicon (450 nucleotides). Amplicons were all sequenced (2x300bp) in a single run on the MiSeq system following the manufacturer’s guidelines.

### Sequencing data analysis

All NGS reads were processed using R 3.6.3 and the dada2 Bioconductor package [58]. Sequences were trimmed and filtered in order to tolerate a maximum of 3 expected errors per paired ends read. Amplicon Sequence Variants (ASV) were inferred using the high-resolution DADA2 method, which distinguishes sequencing errors from real biological variation. Chimeras and low abundance ASV making up < 0.002% of reads were subsequently removed from the data set [59]. Taxonomy was assigned with a naive Bayesian classifier implemented in the DADA2 package and using the Silva (v138) training set. Sequencing data were rarefied to adjust for library size differences across samples (at sequencing depth of 30.000 and 10.000 for experiments conducted in *ApoE*−/− and C57Bl/6 mice, respectively) using the phyloseq Bioconductor package.

The α-diversity indexes (richness, Simpson, and Shannon) in the different microbial communities were calculated using the phyloseq Bioconductor package. The impact of DEP dose on α-diversity indexes was assessed through linear models with DEP dose introduced as a continuous variable. To investigate differences in community composition (β-diversity), weighted UniFrac distances were calculated and plotted using principal coordinates analysis with the GUniFrac R package. The linear effect of DEP dose on β-diversity was assessed through a permutational multivariate analysis of variance (PERMANOVA) with DEP dose introduced as a continuous variable, using the ADONIS function of the vegan package in R.

To identify the bacterial genera, families and phyla impacted by DEP exposure, ASVs were grouped at the genus, family, and phylum level. The counts were then normalized to their relative abundance before applying centered log-ratio (CLR) transformation for compositional data, as described previously [60]. Linear models were built on the transformed data with DEP dose introduced as a continuous variable. When the effect of DEP dose was assessed on multiple outcomes (e.g. relative abundance of several families), *p*-values were corrected (q-values) using the Benjamini-Hochberg [61] methodology to control the false discovery rate (FDR) at 10%.

### Quantification of SCFA

SCFA were extracted from cecal content according to Garcia-Villalba et al. [62]. Approximately 80 mg cecal content were vortexed for 2 min in 1.7 ml formic acid 9% v/v and then centrifuged 10 min at 17900 *g*. Ethyl acetate (350 μl) was added to the supernatant (1.5 ml), vortexed for 2 min and then centrifuged 10 min at 17900 *g*. An internal standard (10 μl methyl valeric acid 500 μM, Sigma Aldrich) was added to 100 μl of the organic phase. Acetic, propionic and butyric acid (Sigma) standards solutions (200 mM) were mixed, serially diluted and extracted as for the cecal content samples. Ten μl internal standard was also added to the standards. Standards and samples were analyzed by gas chromatography-mass spectrometry on a GC HP 6890 (Agilent Technologies, Santa Clara, USA) coupled with a MSD 5973 (Agilent Technologies) with the HpChem software (Agilent Technologies). Results expressed per wet weight of cecal content were normalized to the mean of the respective controls. As DEP are known to adsorb several chemicals, we verified that SCFA were not quenched by DEP in cecal content (Fig. S13).

### Statistics

With the exception of sequencing data, graphs and statistical analyses were computed with GraphPad Prism 8.3.1 (GraphPad Software, San Diego, USA). All results are expressed as means ± standard error on the mean (SEM). Differences between control and treated groups were evaluated by analysis of variance (ANOVA) followed by a Dunnett’s multiple comparison and/or a test for linear trend as appropriate. Statistical significance was considered at *P* < 0.05 and Q < 0.10.

## Supplementary Information

**Additional file 1; Fig. S1.** Effect of gastrointestinal DEP exposure on blood cell counts. White blood cells (A, E), lymphocytes (B, F), monocytes (C, G), neutrophils (D, H) and red blood cells (I) counts in *ApoE*^−/−^ (A-D) and C57BL/6 (E-I) mice treated 3x/week with DEP during 3 or 6 months, respectively,. Data were analyzed by a one-way ANOVA followed by a Dunnett’s test (* *p* ≤ 0.05, ** *p* ≤ 0.01) and a test for linear trend (indicated *p* values) (horizontal bars indicate the means, *n* = 8 for *ApoE*^−/−^, *n* = 10 for C57BL/6). **Fig. S2.** Effect of gastrointestinal DEP exposure on plasma liver enzymes AST (A, C) and ALT (B, D) were measured in the plasma of *ApoE*^−/−^ (A, B) and C57BL/6 (C, D) treated 3x/week during 3 or 6 months, respectively, with PBS (CTL), 40, 200 or 1000 ng DEP/gavage. Data were analyzed by a one-way ANOVA followed by a Dunnett’s test and a test for linear trend (n = 8 for *ApoE*^−/−^, n = 10 for C57BL/6). **Fig. S3.** Histological analysis of ileum and colonic mucosa of *ApoE*^−/−^ mice treated with DEP. Tissue was collected from *ApoE*^−/−^ mice gavaged with DEP during 3 months. Sections were stained with hematoxylin and eosin. Ileum (A, C, E) and colon (B, D, F) in controls (A, B) or mice treated with 200 ng/gavage (C, D) or 1000 ng/gavage (E, F) 3 times per week. Magnification 100x (bar, 100 μm), inserts 800x. **Fig. S4.** Histological analysis of ileum and colonic mucosa of C57BL/6 mice treated with DEP. Tissue was collected from C57BL/6 mice gavaged with DEP during 6 months. Sections were stained with hematoxylin and eosin. Ileum (A, D, E, G) and colon (B, D, F, H) in controls (A,B) or mice treated with 40 ng/gavage (C,D), 200 ng/gavage (E,F) or 1000 ng/gavage (G,H) 3 times per week. Magnification 100x (bar, 100 μm), inserts 800x. **Fig. S5.** Effect of DEP exposure on the relative abundance of phyla in *ApoE* ^−/−^ mice. The relative abundance of bacteria was calculated based on ASVs and taxonomic classification derived from SILVA database. (A) Overview of the relative abundance of bacteria depicted at the phylum level in mice exposed to vehicle control, 200 and 1000 ng DEP/gavage. **Fig. S6.** Effect of DEP exposure on the relative abundance of family in *ApoE* ^−/−^ mice. The relative abundance of bacteria was calculated based on ASVs and taxonomic classification derived from SILVA database. (A) Overview of the average relative abundance of bacteria depicted at the family level in groups of mice exposed to vehicle control, 200 or 1000 ng DEP/gavage. (B) Relative abundance of selected family was plotted against the dose (Q-values for linear dose effect, *n* = 8). **Fig. S7.** Effect of DEP exposure on the relative abundance of genera in *ApoE* ^−/−^ mice. The relative abundance of bacteria was calculated based on ASVs and taxonomic classification derived from SILVA database. Overview of the average relative abundance of bacteria depticted at the genus level in groups of mice exposed to vehicle control, 200 or 1000 ng DEP/gavage. **Fig. S8.** Effect of DEP exposure on the relative abundance of phyla in C57BL/6 mice. The relative abundance of bacteria was calculated based on ASVs and taxonomic classification derived from SILVA database. (A) Overview of the average relative abundance of bacteria depicted at the phylum level in groups of mice exposed to vehicle control, 40, 200 or 1000 ng DEP/gavage. (B–E) Relative abundance of selected phyla was plotted against the dose (Q-values for linear dose effect, *n* = 10).**Fig. S9.** Effect of DEP exposure on the relative abundance of family in C57BL/6 mice. The relative abundance of bacteria was calculated based on ASVs and taxonomic classification derived from SILVA database. (A) Overview of the average relative abundance of bacteria depicted at the family level in groups of mice exposed to vehicle control, 40, 200 or 1000 ng DEP/gavage. (B–C) Relative abundance of selected family was plotted against the dose (Q-values for linear dose effect, n = 10). **Fig. S10.** Effect of DEP exposure on the relative abundance of genera in C57BL/6 mice. The relative abundance of bacteria at genus level was calculated based on ASVs and taxonomic classification derived from SILVA database. (A) Overview of the average relative abundance of bacteria depicted at the genus level in groups of mice exposed to vehicle control, 40, 200 or 1000 ng DEP/gavage. (B–D) Relative abundance of selected genus was plotted against the dose (Q-values for linear dose effect, n = 10). **Fig. S11.** Cardiovascular effects of gastrointestinal DEP exposure. Sections of abdominal aorta (H&E staining) from *ApoE*^−/−^ mice: controls (A) or treated with 200 (B) or 1000 ng/gavage (C), and from C57BL/6 mice: controls (D) or treated with 40 (E), 200 (F) or 1000 ng/gavage (G). Magnification 100x (bar size 100 μm), inserts 800x. **Fig. S12.** Hydrodynamic mean diameter of SRM2795. The hydrodynamic mean diameter (Z-average) of SRM2795 was measured by DLS on a 5.15 μg/ml suspension in PBS after sonication (concentration of DEP in the suspension used to deliver 1000 ng/gavage). **Fig. S13.** DEP do not quench SCFA in cecal content. The cecal content of five naive C57Bl/6 mice were pooled, and aliquoted in fractions of about 80 mg to which we added 0, 5, 25 or 125 ng DEP. These samples were carefully mixed, and analyzed for SCFA by LC-MS (*p*-value for ANOVA; *n* = 6 technical replicates). The mouse cecal content (200 mg) corresponds on average to 5% of the daily food intake (4000 mg). Thus, the highest spiked amount (125 ng DEP/80 mg cecal content, equivalent to 312.5 ng DEP per cecum) corresponds to a daily intake of 6250 ng DEP, which exceeds the highest gavage dose 1000 ng DEP used in the present study.

## Data Availability

The metagenomics datasets generated and analyzed during the current study are available in the European Nucleotide Archive repository under study PRJEB39619 and PRJEB39623.

## Abbreviations

ALT: Alanine aminotransferase
ANOVA: Analysis of variance
AST: Aspartate aminotransferase
b.w.: Body weight
CM: Cardiometabolic
CV: Cardiovascular
DEP: Diesel exhaust particles
GM: Gut microbiota
H&E: Hematoxylin-eosin
HDL: High density lipoprotein
NIST: National Institute of Standards and Technology
NP: Nanoparticles
PBS: Phosphate-buffered solution
PM: Particulate matter
SCFA: Short chain fatty acid
SEM: Standard error on the mean
UFP: Ultrafine particles
Z-average: Hydrodynamic mean diameter
w.t.: Wild-type
