# Supplementary material for: Diesel exhaust particles alter the profile and function of the gut microbiota upon subchronic oral administration in mice
Source: Part Fibre Toxicol. 2021 Feb 9;18:7. doi: 10.1186/s12989-021-00400-7 (PMC7871568; doi:10.1186/s12989-021-00400-7)
Supplement: Supplementary file 1 — Additional file 1; Fig. S1. Effect of gastrointestinal DEP exposure on blood cell counts. White blood cells (A, E), lymphocytes (B, F), monocytes (C, G), neutrophils (D, H) and red blood cells (I) counts in ApoE−/− (A-D) and C57BL/6 (E-I) mice treated 3x/week with DEP during 3 or 6 months, respectively,. Data were analyzed by a one-way ANOVA followed by a Dunnett’s test (* p ≤ 0.05, ** p ≤ 0.01) and a test for linear trend (indicated p values) (horizontal bars indicate the means, n = 8 for ApoE−/−, n = 10 for C57BL/6). Fig. S2. Effect of gastrointestinal DEP exposure on plasma liver enzymes AST (A, C) and ALT (B, D) were measured in the plasma of ApoE−/− (A, B) and C57BL/6 (C, D) treated 3x/week during 3 or 6 months, respectively, with PBS (CTL), 40, 200 or 1000 ng DEP/gavage. Data were analyzed by a one-way ANOVA followed by a Dunnett’s test and a test for linear trend (n = 8 for ApoE−/−, n = 10 for C57BL/6). Fig. S3. Histological analysis of ileum and colonic mucosa of ApoE−/− mice treated with DEP. Tissue was collected from ApoE−/− mice gavaged with DEP during 3 months. Sections were stained with hematoxylin and eosin. Ileum (A, C, E) and colon (B, D, F) in controls (A, B) or mice treated with 200 ng/gavage (C, D) or 1000 ng/gavage (E, F) 3 times per week. Magnification 100x (bar, 100 μm), inserts 800x. Fig. S4. Histological analysis of ileum and colonic mucosa of C57BL/6 mice treated with DEP. Tissue was collected from C57BL/6 mice gavaged with DEP during 6 months. Sections were stained with hematoxylin and eosin. Ileum (A, D, E, G) and colon (B, D, F, H) in controls (A,B) or mice treated with 40 ng/gavage (C,D), 200 ng/gavage (E,F) or 1000 ng/gavage (G,H) 3 times per week. Magnification 100x (bar, 100 μm), inserts 800x. Fig. S5. Effect of DEP exposure on the relative abundance of phyla in ApoE −/− mice. The relative abundance of bacteria was calculated based on ASVs and taxonomic classification derived from SILVA database. (A) Overview of the relative abundance [file 12989_2021_400_MOESM1_ESM.pptx]

## Slide 1
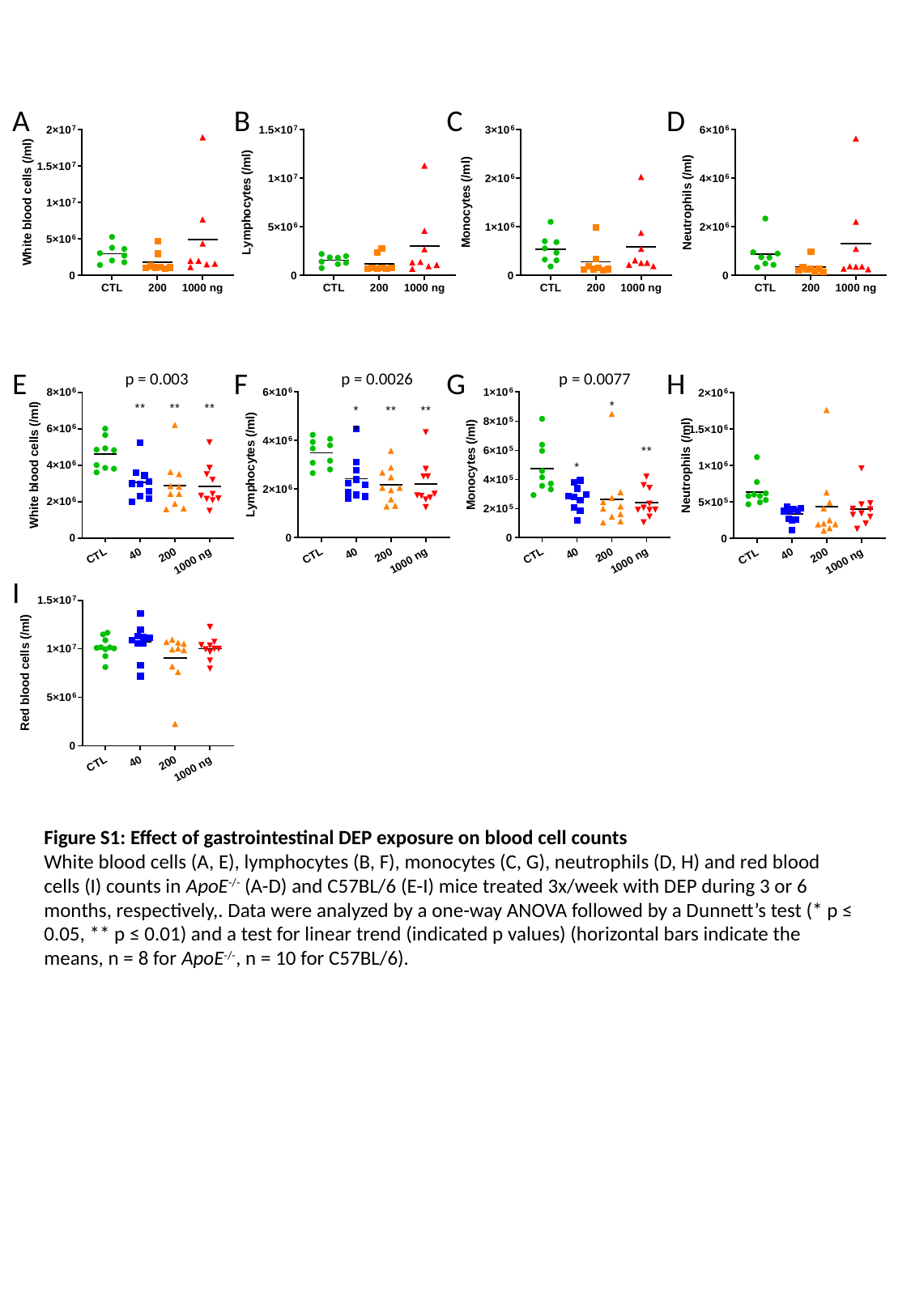

D
A
B
C
H
E
F
G
p = 0.003
p = 0.0026
p = 0.0077
*
I
Figure S1: Effect of gastrointestinal DEP exposure on blood cell counts
White blood cells (A, E), lymphocytes (B, F), monocytes (C, G), neutrophils (D, H) and red blood cells (I) counts in ApoE-/- (A-D) and C57BL/6 (E-I) mice treated 3x/week with DEP during 3 or 6 months, respectively,. Data were analyzed by a one-way ANOVA followed by a Dunnett’s test (* p ≤ 0.05, ** p ≤ 0.01) and a test for linear trend (indicated p values) (horizontal bars indicate the means, n = 8 for ApoE-/-, n = 10 for C57BL/6).

## Slide 2
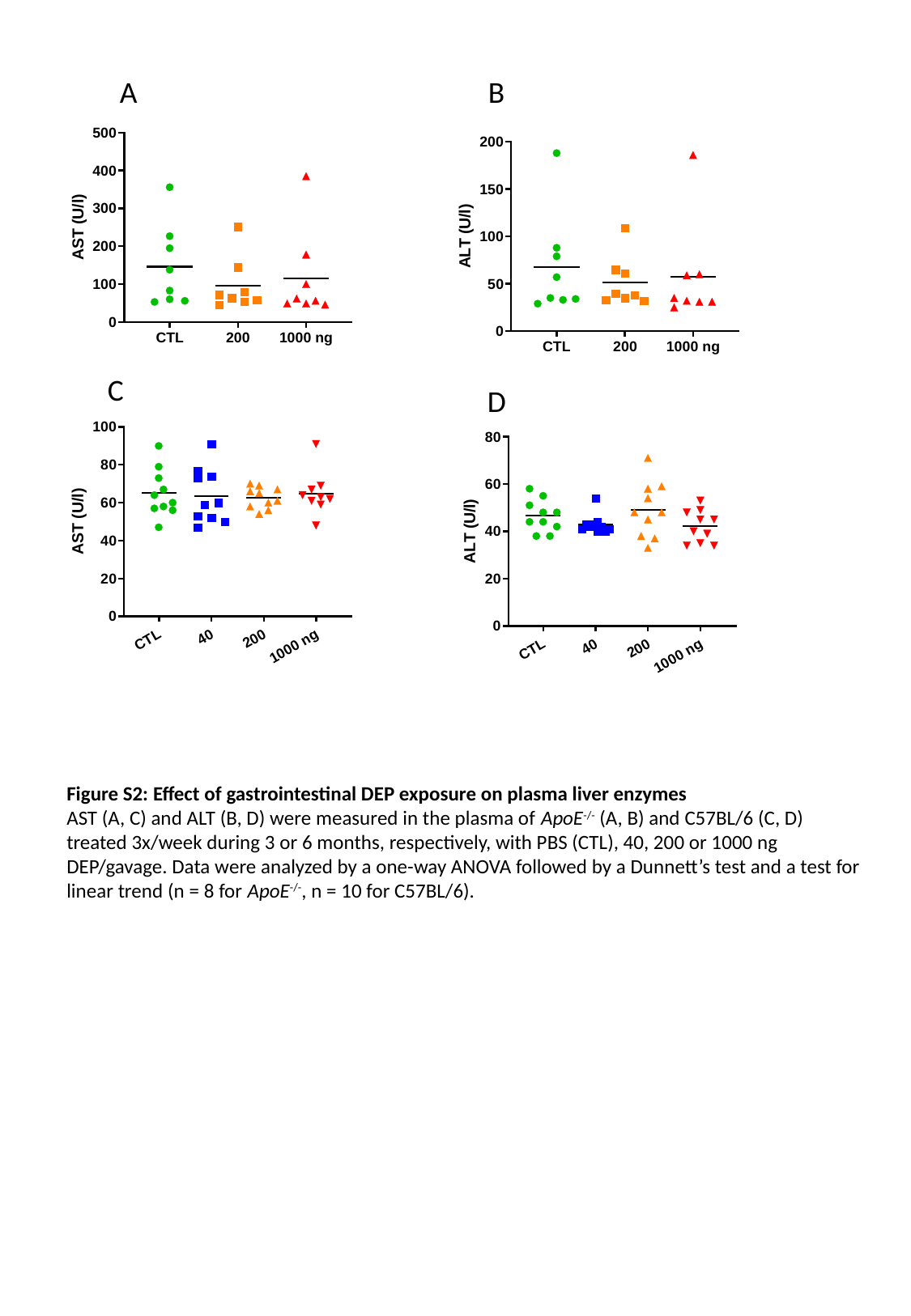

A
B
C
D
Figure S2: Effect of gastrointestinal DEP exposure on plasma liver enzymes
AST (A, C) and ALT (B, D) were measured in the plasma of ApoE-/- (A, B) and C57BL/6 (C, D) treated 3x/week during 3 or 6 months, respectively, with PBS (CTL), 40, 200 or 1000 ng DEP/gavage. Data were analyzed by a one-way ANOVA followed by a Dunnett’s test and a test for linear trend (n = 8 for ApoE-/-, n = 10 for C57BL/6).

## Slide 3
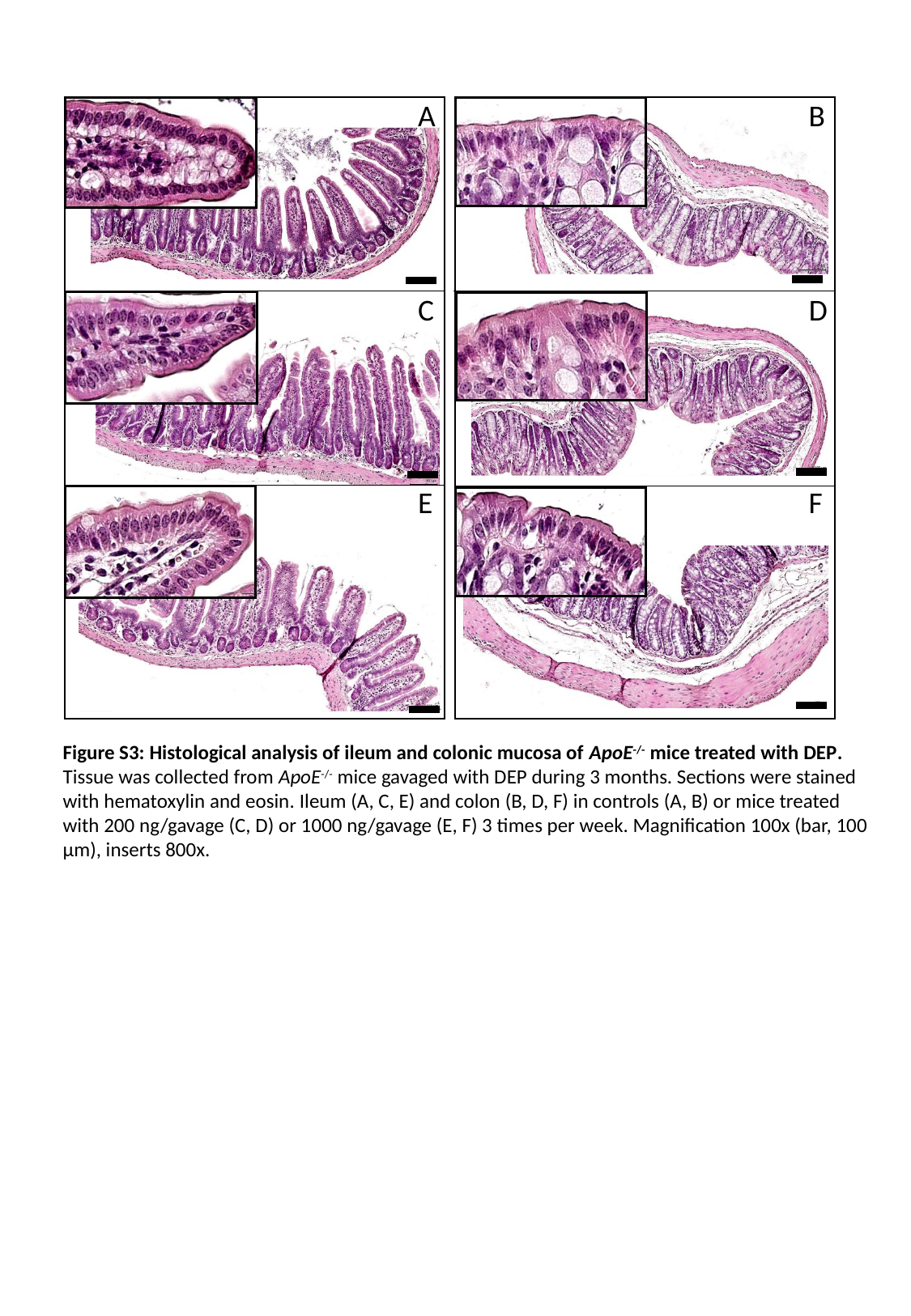

A
B
C
D
E
F
Figure S3: Histological analysis of ileum and colonic mucosa of ApoE-/- mice treated with DEP.
Tissue was collected from ApoE-/- mice gavaged with DEP during 3 months. Sections were stained with hematoxylin and eosin. Ileum (A, C, E) and colon (B, D, F) in controls (A, B) or mice treated with 200 ng/gavage (C, D) or 1000 ng/gavage (E, F) 3 times per week. Magnification 100x (bar, 100 µm), inserts 800x.

## Slide 4
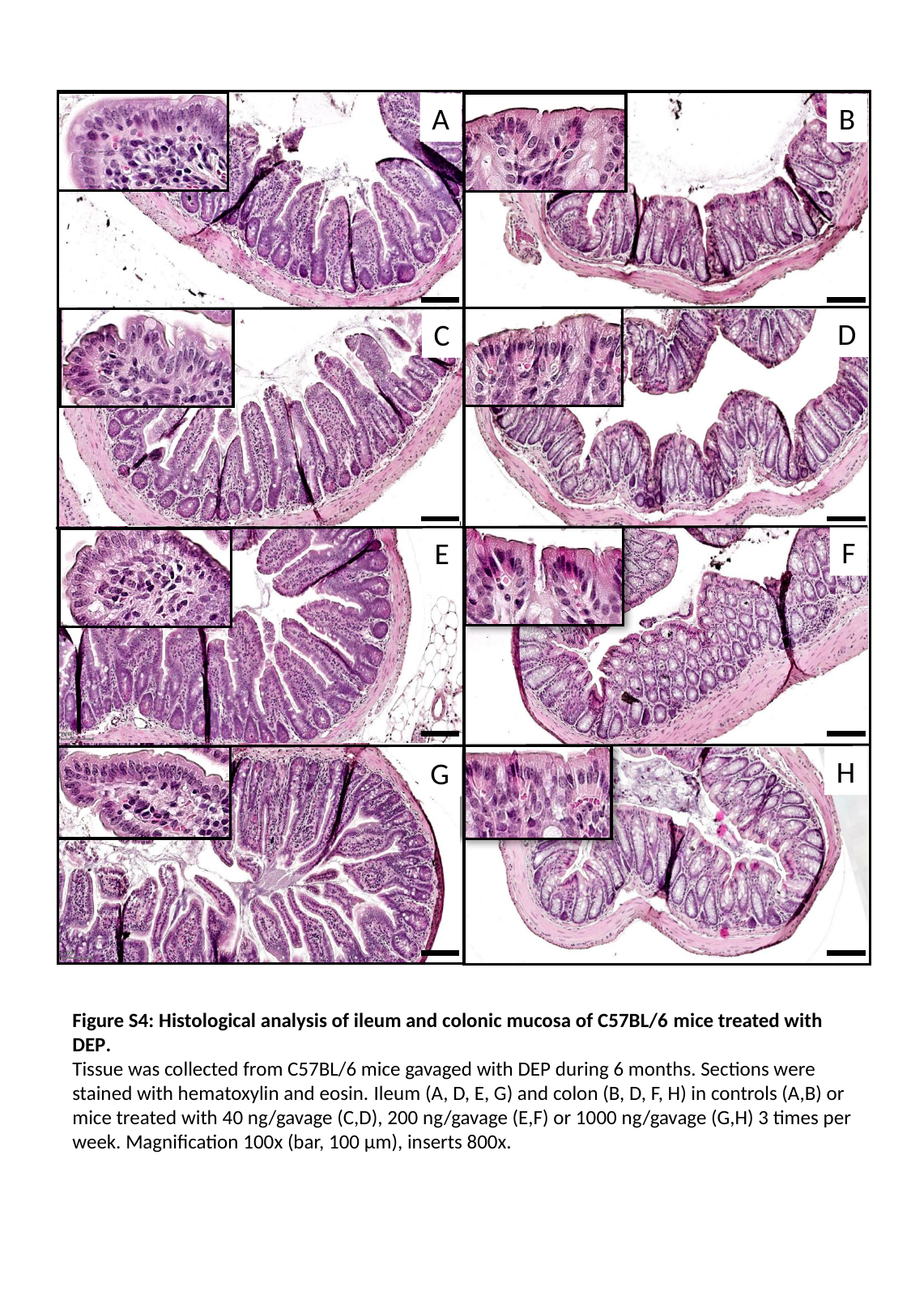

A
B
D
C
F
E
H
G
Figure S4: Histological analysis of ileum and colonic mucosa of C57BL/6 mice treated with DEP.
Tissue was collected from C57BL/6 mice gavaged with DEP during 6 months. Sections were stained with hematoxylin and eosin. Ileum (A, D, E, G) and colon (B, D, F, H) in controls (A,B) or mice treated with 40 ng/gavage (C,D), 200 ng/gavage (E,F) or 1000 ng/gavage (G,H) 3 times per week. Magnification 100x (bar, 100 µm), inserts 800x.

## Slide 5
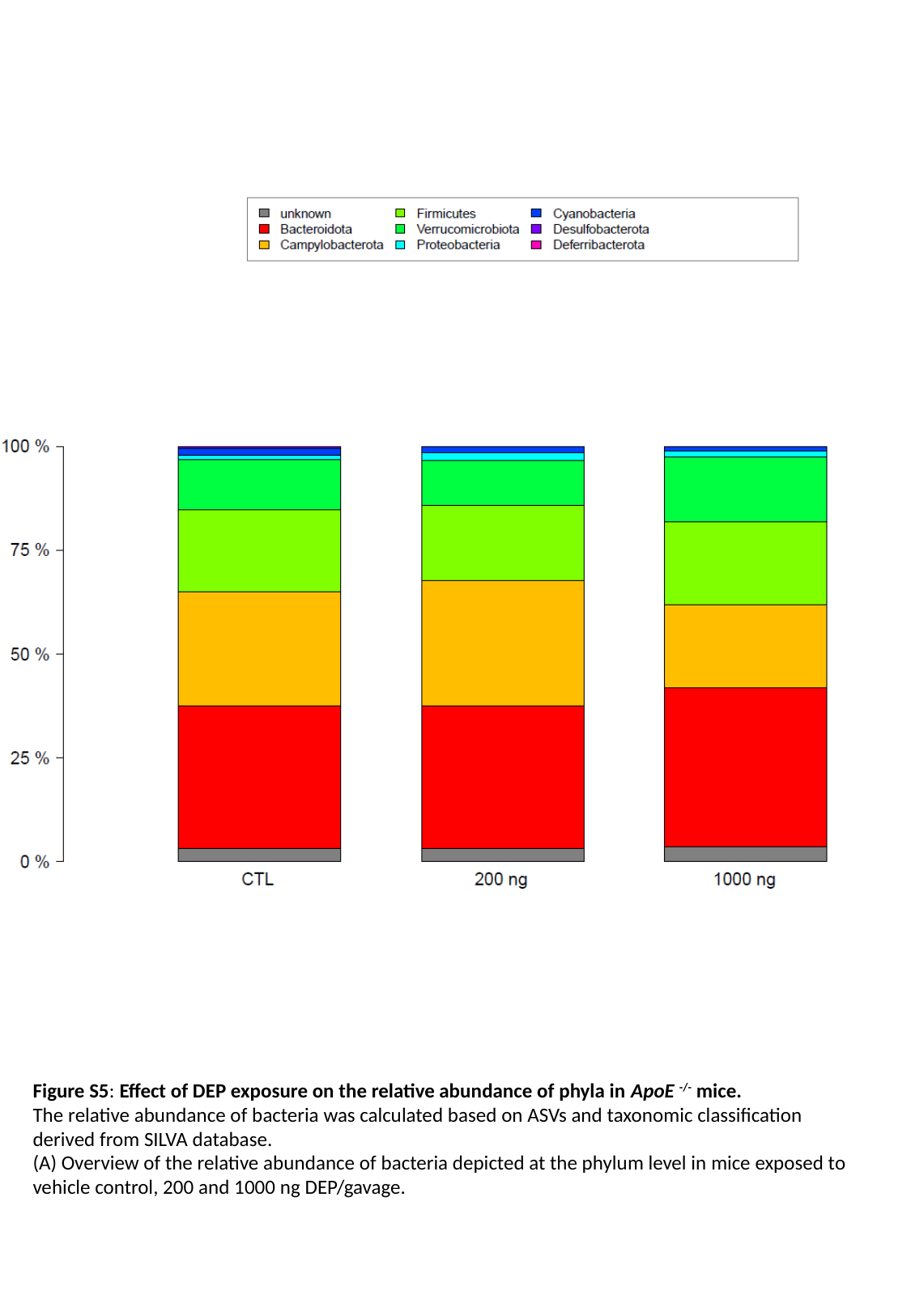

Figure S5: Effect of DEP exposure on the relative abundance of phyla in ApoE -/- mice.
The relative abundance of bacteria was calculated based on ASVs and taxonomic classification derived from SILVA database.
(A) Overview of the relative abundance of bacteria depicted at the phylum level in mice exposed to vehicle control, 200 and 1000 ng DEP/gavage.

## Slide 6
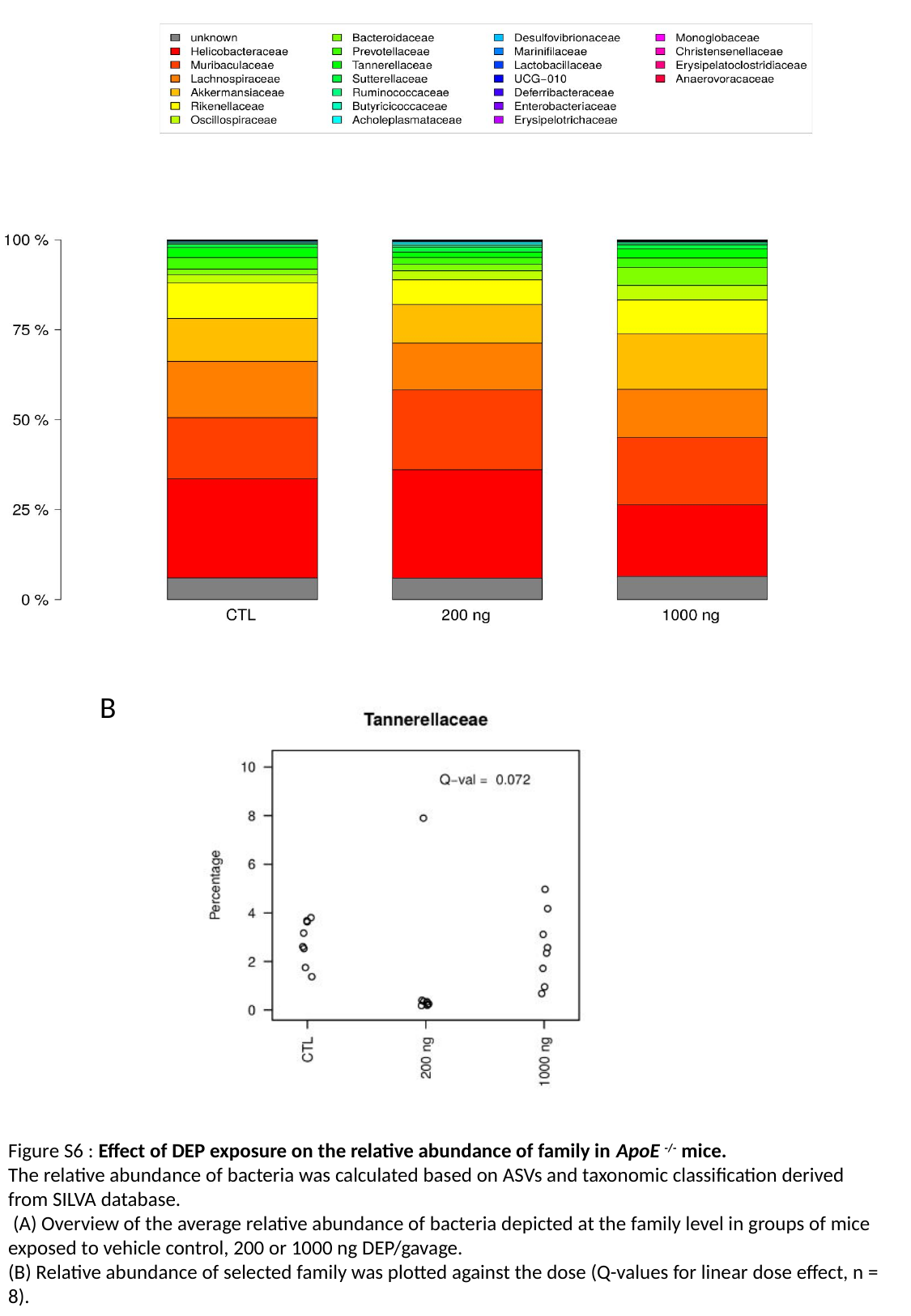

A
B
Figure S6 : Effect of DEP exposure on the relative abundance of family in ApoE -/- mice.
The relative abundance of bacteria was calculated based on ASVs and taxonomic classification derived from SILVA database.
 (A) Overview of the average relative abundance of bacteria depicted at the family level in groups of mice exposed to vehicle control, 200 or 1000 ng DEP/gavage.
(B) Relative abundance of selected family was plotted against the dose (Q-values for linear dose effect, n = 8).

## Slide 7
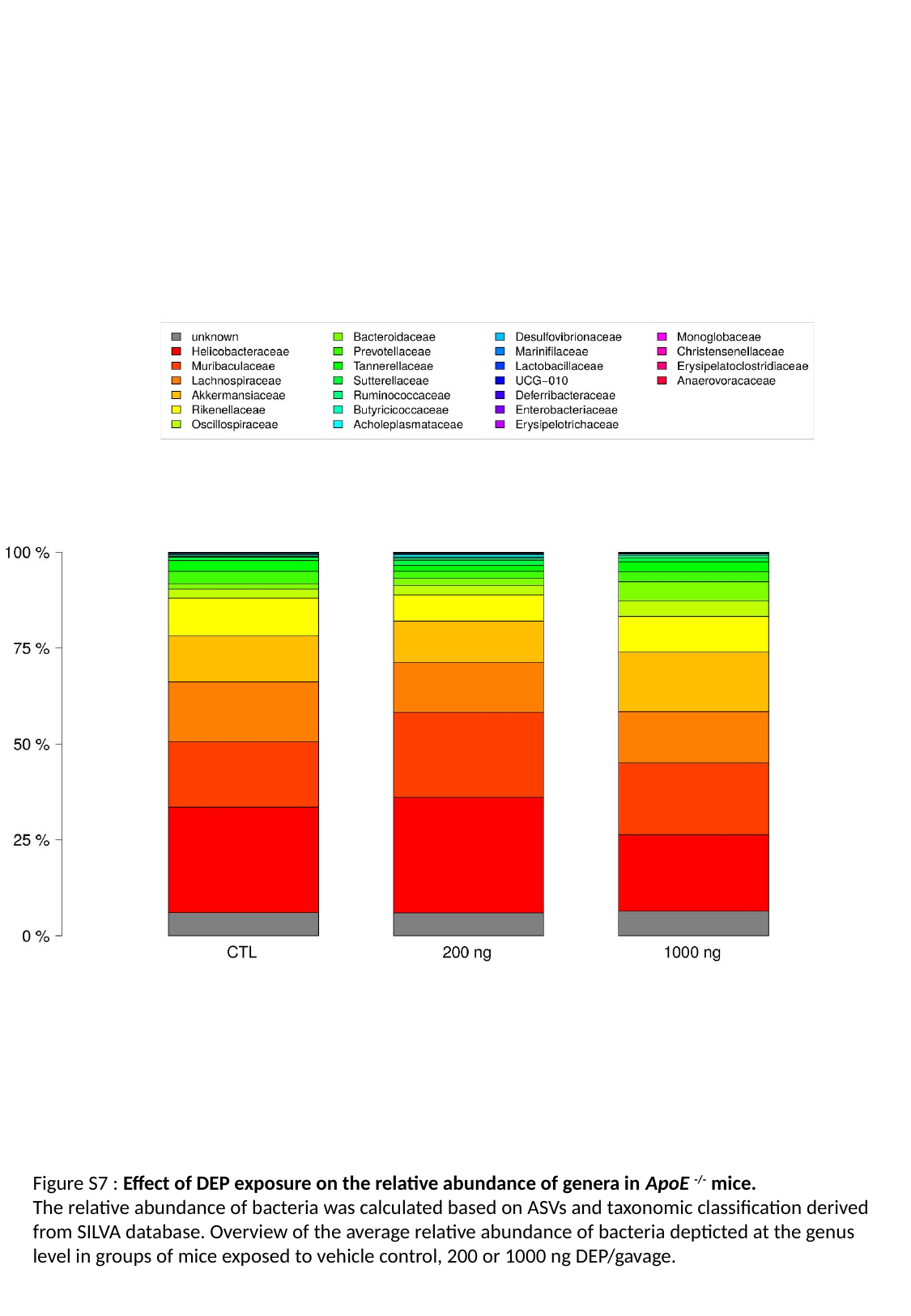

Figure S7 : Effect of DEP exposure on the relative abundance of genera in ApoE -/- mice.
The relative abundance of bacteria was calculated based on ASVs and taxonomic classification derived from SILVA database. Overview of the average relative abundance of bacteria depticted at the genus level in groups of mice exposed to vehicle control, 200 or 1000 ng DEP/gavage.

## Slide 8
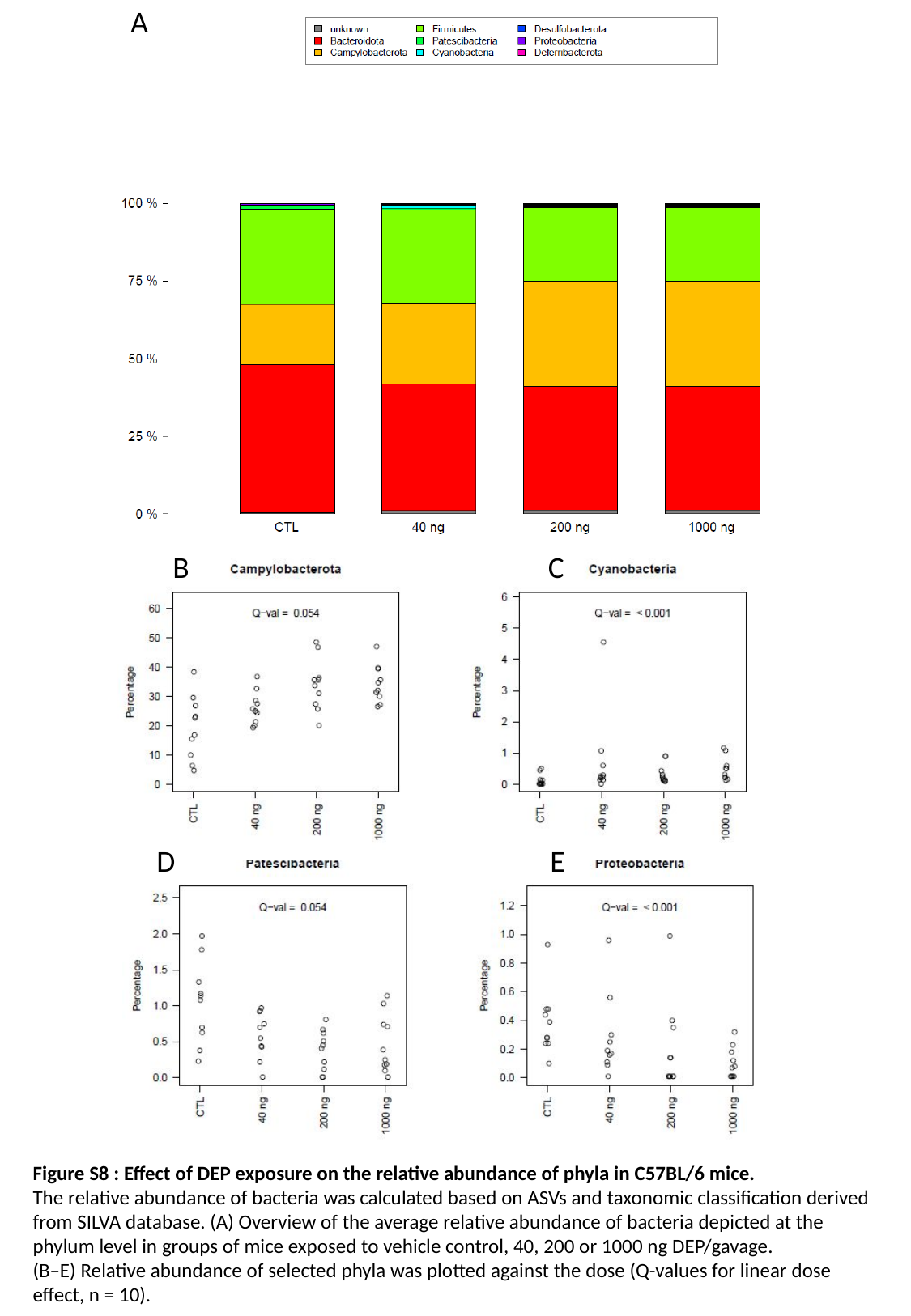

A
B
C
D
E
Figure S8 : Effect of DEP exposure on the relative abundance of phyla in C57BL/6 mice.
The relative abundance of bacteria was calculated based on ASVs and taxonomic classification derived from SILVA database. (A) Overview of the average relative abundance of bacteria depicted at the phylum level in groups of mice exposed to vehicle control, 40, 200 or 1000 ng DEP/gavage.
(B–E) Relative abundance of selected phyla was plotted against the dose (Q-values for linear dose effect, n = 10).

## Slide 9
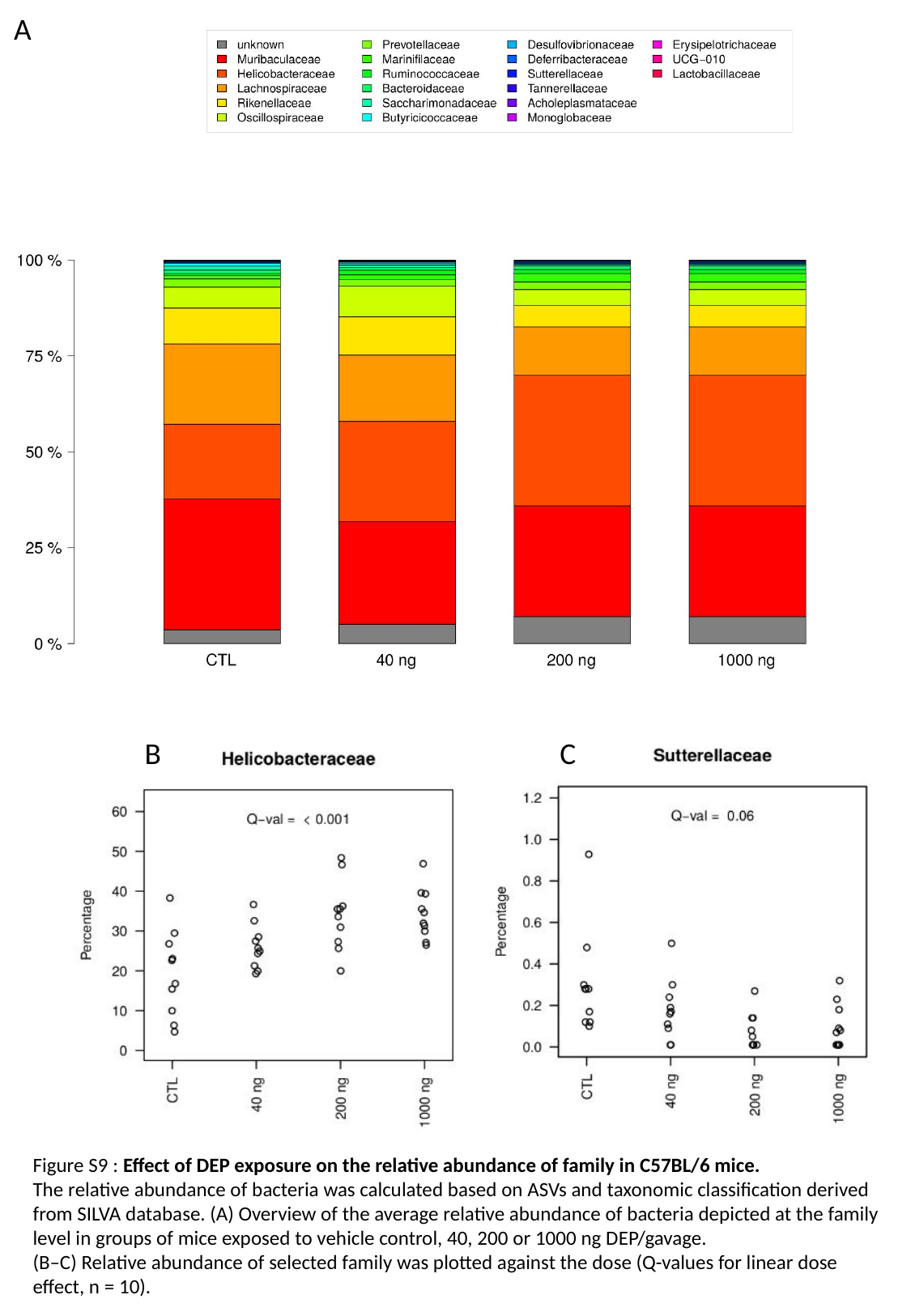

A
B
C
Figure S9 : Effect of DEP exposure on the relative abundance of family in C57BL/6 mice.
The relative abundance of bacteria was calculated based on ASVs and taxonomic classification derived from SILVA database. (A) Overview of the average relative abundance of bacteria depicted at the family level in groups of mice exposed to vehicle control, 40, 200 or 1000 ng DEP/gavage.
(B–C) Relative abundance of selected family was plotted against the dose (Q-values for linear dose effect, n = 10).

## Slide 10
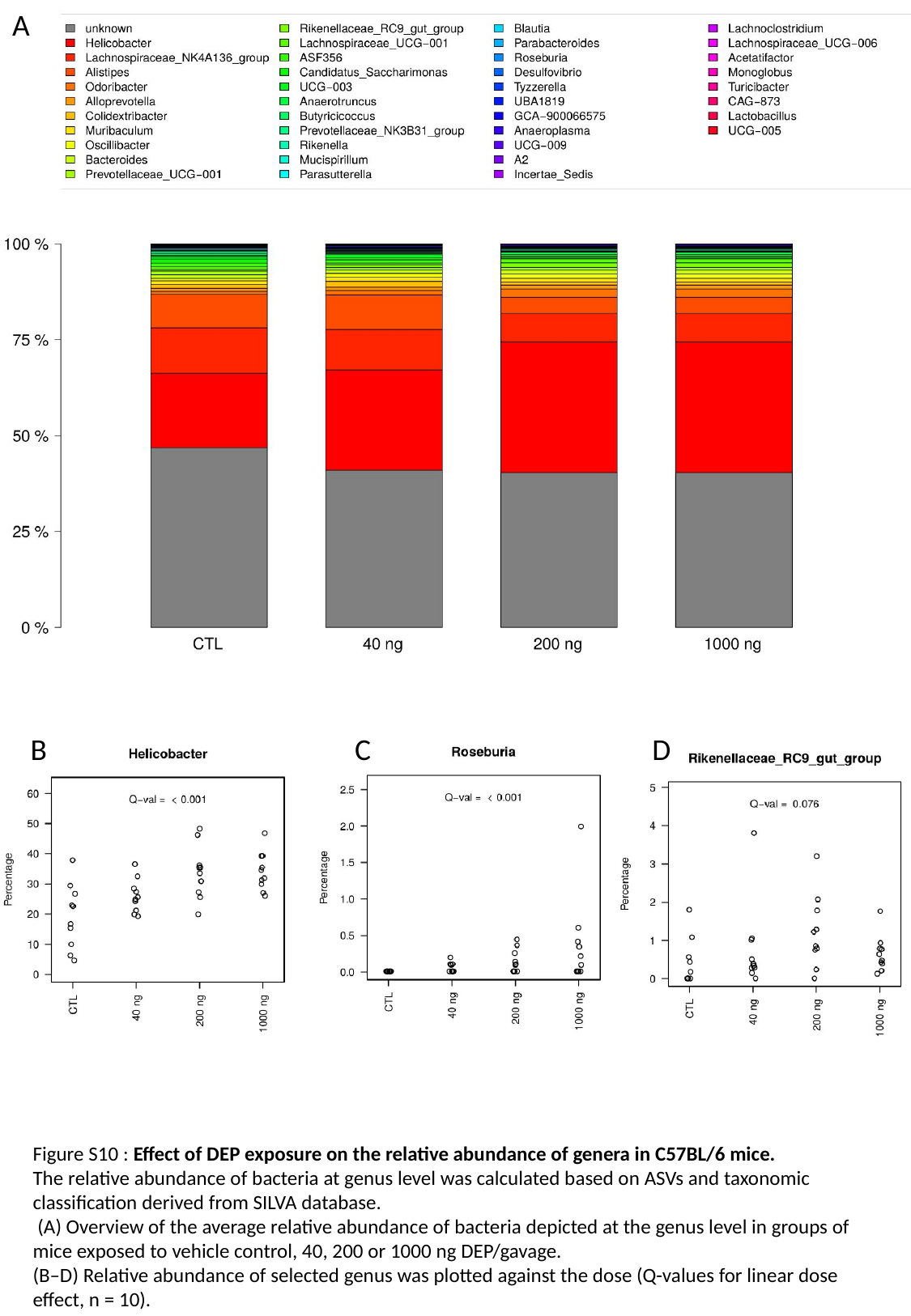

A
B
C
D
Figure S10 : Effect of DEP exposure on the relative abundance of genera in C57BL/6 mice.
The relative abundance of bacteria at genus level was calculated based on ASVs and taxonomic classification derived from SILVA database.
 (A) Overview of the average relative abundance of bacteria depicted at the genus level in groups of mice exposed to vehicle control, 40, 200 or 1000 ng DEP/gavage.
(B–D) Relative abundance of selected genus was plotted against the dose (Q-values for linear dose effect, n = 10).

## Slide 11
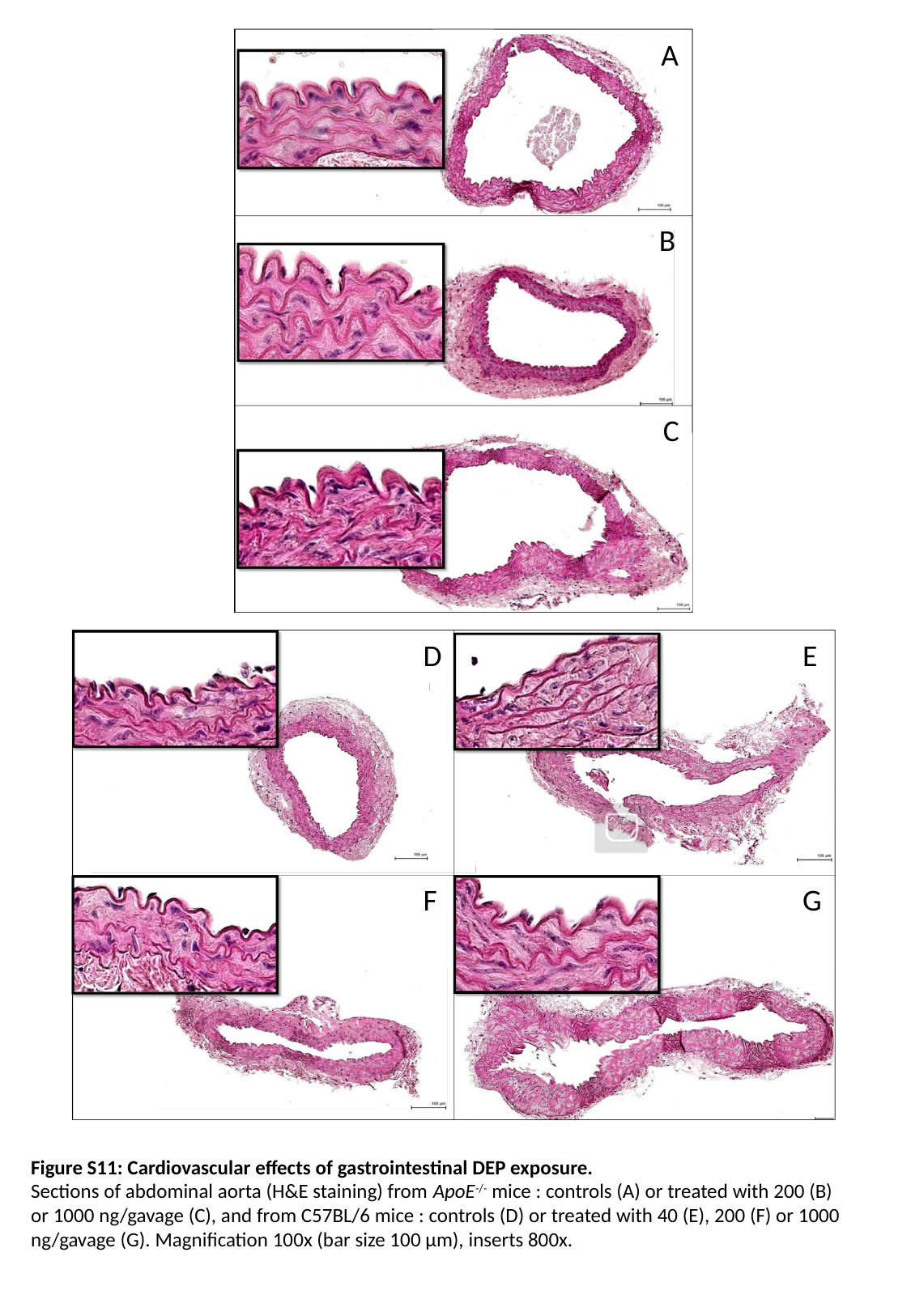

A
B
C
D
E
F
G
Figure S11: Cardiovascular effects of gastrointestinal DEP exposure.
Sections of abdominal aorta (H&E staining) from ApoE-/- mice : controls (A) or treated with 200 (B) or 1000 ng/gavage (C), and from C57BL/6 mice : controls (D) or treated with 40 (E), 200 (F) or 1000 ng/gavage (G). Magnification 100x (bar size 100 µm), inserts 800x.

## Slide 12
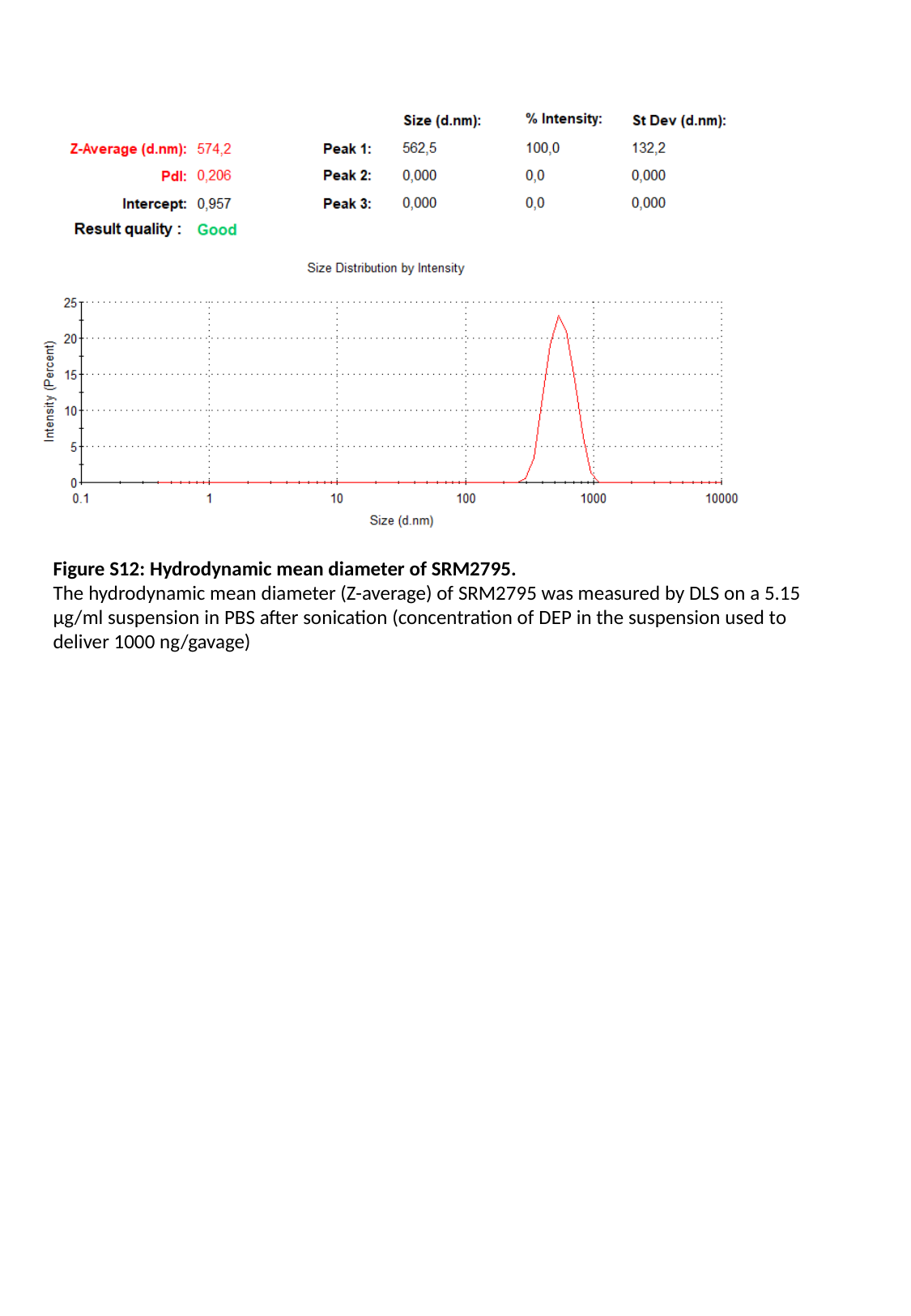

Figure S12: Hydrodynamic mean diameter of SRM2795.
The hydrodynamic mean diameter (Z-average) of SRM2795 was measured by DLS on a 5.15 µg/ml suspension in PBS after sonication (concentration of DEP in the suspension used to deliver 1000 ng/gavage)

## Slide 13
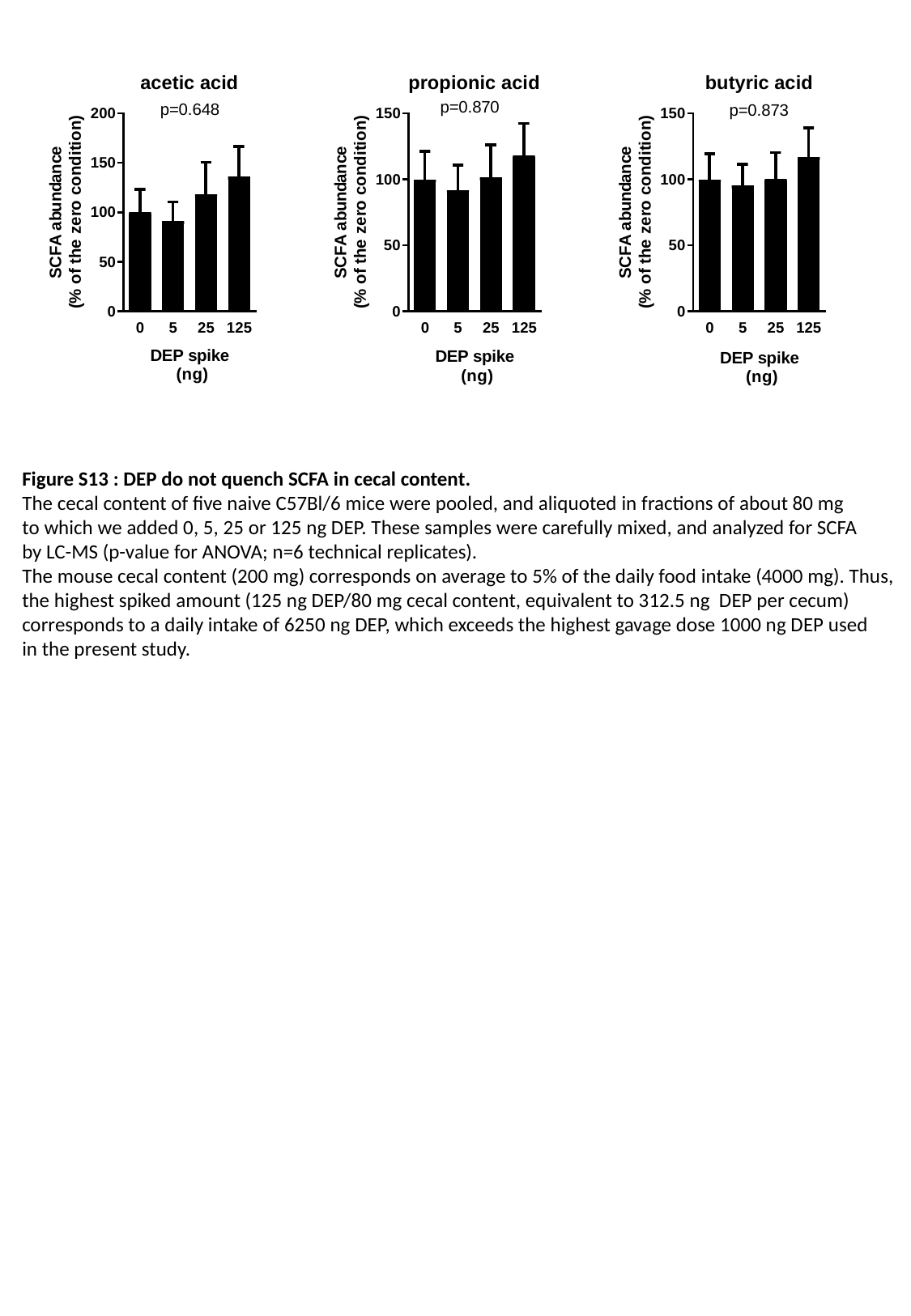

Figure S13 : DEP do not quench SCFA in cecal content.
The cecal content of five naive C57Bl/6 mice were pooled, and aliquoted in fractions of about 80 mg
to which we added 0, 5, 25 or 125 ng DEP. These samples were carefully mixed, and analyzed for SCFA
by LC-MS (p-value for ANOVA; n=6 technical replicates).
The mouse cecal content (200 mg) corresponds on average to 5% of the daily food intake (4000 mg). Thus,
the highest spiked amount (125 ng DEP/80 mg cecal content, equivalent to 312.5 ng DEP per cecum)
corresponds to a daily intake of 6250 ng DEP, which exceeds the highest gavage dose 1000 ng DEP used
in the present study.
